# Isolated Rh atoms in dehydrogenation catalysis

**DOI:** 10.1038/s41598-023-31157-y

**Published:** 2023-03-17

**Authors:** Haiko Wittkämper, Rainer Hock, Matthias Weißer, Johannes Dallmann, Carola Vogel, Narayanan Raman, Nicola Taccardi, Marco Haumann, Peter Wasserscheid, Tzung-En Hsieh, Sven Maisel, Michael Moritz, Christoph Wichmann, Johannes Frisch, Mihaela Gorgoi, Regan G. Wilks, Marcus Bär, Mingjian Wu, Erdmann Spiecker, Andreas Görling, Tobias Unruh, Hans-Peter Steinrück, Christian Papp

**Affiliations:** 1 Lehrstuhl für Physikalische Chemie II, Friedrich-Alexander-Universität Erlangen-Nürnberg (FAU), Egerlandstr. 3, 91058 Erlangen, Germany; 2 Lehrstuhl für Kristallographie und Strukturphysik, Friedrich-Alexander-Universität Erlangen-Nürnberg (FAU), Staudtstr. 3, 91058 Erlangen, Germany; 3 Lehrstuhl für Chemische Reaktionstechnik (CRT), Friedrich-Alexander-Universität Erlangen-Nürnberg (FAU), Egerlandstr. 3, 91058 Erlangen, Germany; 4 Forschungszentrum Jülich GmbH, Helmholtz-Institute Erlangen-Nürnberg for Renewable Energy (IEK-11), Egerlandstr. 3, 91058 Erlangen, Germany; 5 Department Interface Design, Helmholtz-Zentrum Berlin für Materialien und Energie GmbH (HZB), 12489 Berlin, Germany; 6 Energy Materials In-Situ Laboratory Berlin (EMIL), HZB, 12489 Berlin, Germany; 7 Lehrstuhl für Theoretische Chemie, Friedrich-Alexander-Universität Erlangen-Nürnberg (FAU), Egerlandstr. 3, 91058 Erlangen, Germany; 8 Department X-Ray Spectroscopy at Interfaces of Thin Films, Helmholtz Institute for Renewable Energy (HI ERN), 12489 Berlin, Germany; 9 Lehrstuhl für Werkstoffwissenschaften (Mikro- und Nanostrukturforschung), Cauerstraße 3, 91058 Erlangen, Germany; 10 Physikalische und Theoretische Chemie, Freie Universität Berlin, Arnimallee 22, 14195 Berlin, Germany

**Keywords:** Catalysis, Materials chemistry, Physical chemistry, Surface chemistry, Theoretical chemistry

## Abstract

Isolated active sites have great potential to be highly efficient and stable in heterogeneous catalysis, while enabling low costs due to the low transition metal content. Herein, we present results on the synthesis, first catalytic trials, and characterization of the Ga_9_Rh_2_ phase and the hitherto not-studied Ga_3_Rh phase. We used XRD and TEM for structural characterization, and with XPS, EDX we accessed the chemical composition and electronic structure of the intermetallic compounds. In combination with catalytic tests of these phases in the challenging propane dehydrogenation and by DFT calculations, we obtain a comprehensive picture of these novel catalyst materials. Their specific crystallographic structure leads to isolated Rhodium sites, which is proposed to be the decisive factor for the catalytic properties of the systems.

## Introduction

Light olefins like propylene are valuable bulk chemicals for the polymer industry and other chemical sectors with a demand that exceeds the supply. One approach to close this demand–supply gap for propylene is the catalytic dehydrogenation of propane^1^. This process yields two valuable products: hydrogen and propylene, but is highly challenging because of rapid catalyst deactivation due to coke formation and the endothermic nature of the reaction^2,3^. However, in a number of recent studies effective dehydrogenation catalysts with long lifetimes have been presented^4–9^. In all cases, the underlying design principles of these catalysts are the same: deactivation and activity in hydrogenation and dehydrogenation reactions strongly depend on the size and arrangement of the atom ensembles of the active centers^10^. Isolated single atoms are considered to be the ideal reaction sites, with the challenge of keeping them stable under reaction conditions.

Several approaches to synthesize heterogeneous catalysts with defined isolated reaction sites for dehydrogenation reactions have been proposed. One concept uses solid single atom alloys, that is, substitutional alloys^6,11,12^. In addition, also liquid catalysts in form of Gallium alloys with low Rhodium content (typically below 4 at.%) on an oxidic support have proven to be highly effective in propane dehydrogenation. The latter catalyst systems are denoted as Supported Catalytically Active Liquid Metal Solutions (SCALMS)^4,5^. Thereby, diluting Rhodium in liquid Gallium improves the catalyst stability substantially, due to the separation of the active sites on the surface of the liquid alloy. The liquid nature of these systems guarantees highly uniform single reactions sites, which result in high selectivity.

Another outstanding approach to provide stable and defined site isolation in heterogeneous catalysts are intermetallic compounds, which have been studied quite extensively and have been reviewed recently^13,14^. In many cases, they show improved catalytic properties compared to the pure transition metals. For example, GaPd-based intermetallic compounds have significant covalent contributions to the bonding. This gives rise to very complex structures, in which the transition metal is highly coordinated by the p-block metal. Surfaces of such crystals can contain practically isolated transition metal centers. Regarding their geometry, these are ideal reaction centers, if they remain stable during the reaction^15,16^. Similarly, Ga_1_Pt_1_ intermetallic compounds were shown to be highly selective (99.6%) and stable (operation up to 96 h) for propane dehydrogenation^7^.

Herein, we show how catalytically active Rhodium intermetallic systems that follow the principle of site isolation can be prepared and characterized. Thereby, it is noteworthy to mention that Rhodium is not a common dehydrogenation catalyst as it rapidly deactivates due to coke formation after strong initial activity. Compared to GaPd, Ga_16_Rh_3_, Ga_21_Rh_4_, Ga_9_Rh_2_ (space group Pc) consists of Ga_9_Rh building blocks that resemble single-capped square antiprismatic Ga polyhedra with one Rh atom at the center^17,18^. For Ga_3_Rh, Schubert et al. initially reported the structure to be isostructural to In_3_Ir^19,20^. The tetragonal space group P-4n2 was ﻿assumed based on Debye–Scherrer powder diffraction data. Later on, Pöttgen et al. re-determined the space group of In_3_Ir to be P4_2_/mnm and refined the structure based on single-crystal data^21^.

Besides site isolation, several GaPd and GaRh systems show a change in the electronic structure of the active transition metal in the form of a charge transfer from Ga to the 4d band of Pd/Rh. This causes a shift of the d-band center further towards larger binding energies below the Fermi level^15,22,23^. For transition metal surfaces, it is generally accepted that the d-band center position and the availability of d-band hole states directly influence adsorption behavior and catalytic properties^24^. For a similar reaction to propane dehydrogenation, i.e., the semihydrogenation of acetylene, which also follows a Horiuti–Polanyi mechanism, a downshift of the d-band center is beneficial for the catalytic properties. In particular, for the ternary Ga_1-x_Sn_x_Pd_2_ intermetallic compounds the activity was directly related to the d-band position with an optimum activity at − 3.04 eV^25^.

The aim of our study was to investigate the potential of GaRh intermetallic compounds as catalysts in dehydrogenation catalysis. In the following, we present our results on the preparation and characterization of two catalytically interesting Ga-rich GaRh intermetallic compounds, Ga_9_Rh_2_ and Ga_3_Rh. The crystal structure observed for Ga_3_Rh has, to the best of our knowledge, not been previously described in the literature.

## Methods

For the synthesis of Ga_9_Rh_2_ and Ga_3_Rh, we weighed in stoichiometric amounts of Rh wire (99.9% Goodfellow) and liquid Ga (99.99999% Sigma-Aldrich) into Al_2_O_3_ crucibles (Almath 13 mm × 10.5 mm). To avoid excessive oxidation induced by heating, the synthesis was performed under vacuum conditions (< 10^–5^ mbar) in a 5.6 kW resistance heated (graphite) vacuum furnace, designed for temperatures up to 1800 °C, see SI. The samples were slowly heated to 1000 °C over the next 5 h. The samples were kept at 1000 °C for 10 min and afterward cooled down to 500 °C at a cooling rate of 0.8 °C per minute. After reaching 500 °C, the samples were kept at this temperature for up to 10 h before cooling to room temperature.

Both synthesized alloys were crushed in an agate mortar to produce a fine powder. Ga_9_Rh_2_ was measured with a Panalytical X’pert powder diffractometer and Ni filtered Cu Kα_1,2_ radiation in Θ–Θ geometry. The diffraction pattern was recorded in an angular range from 15° to 120° 2Θ by an XCelerator detector module. The synthesis of Ga_9_Rh_2_ was verified by a Rietveld refinement based on the published structure data by Boström et al.^18^ to the measured powder pattern. The powder pattern of Ga_3_Rh was measured in Θ–Θ geometry with a Rigaku SmartLab copper target rotating anode monochromatized by a Ge Johansson monochromator. The diffracted signal was detected by a Rigaku HyPix-3000 detector (15°–100° 2Θ). Details on structure solution and refinement are given in the SI^26–28^.

For TEM investigation, the crushed sample powder was supported on a standard 200 mesh Cu TEM grids filled with holey carbon network. The TEM grids were studied using a ThermoFischer Scientific Titan Themis monochromated, double Cs-corrected TEM. The composition of the crystallites was evaluated with energy-dispersive X-ray spectrometry with data collected by a Super-X detector array equipped on the TEM. The results reported are quantified based on the Ga-K and Rh-L family using the well accepted Braon-Powell ionization cross-section model as implemented in the Velox software. Well separated single crystalline particles of 500–2000 nm in size were selected for electron diffraction tomography study. These diffraction patterns were compared with simulated diffraction patterns from the literature structure (in case of Ga_9_Rh_2_) and the structure determined via powder XRD (in case of Ga_3_Rh) as validation.

Density-functional theory calculations were carried out using the *Vienna *Ab Initio* Simulation Package* (VASP) employing the projector augmented wave (PAW) method to represent the atomic cores and a plane wave basis set with a kinetic energy cutoff of 400 eV^29–31^. The functional developed by Perdew, Burke, and Ernzerhof (PBE) was applied to describe exchange–correlation effects^32^. A first-order Methfessel-Paxton smearing with a width of 0.2 eV was chosen for geometry optimizations and a tetrahedron smearing with Blöchl corrections for electronic density of states (DOS) calculations^33,34^. Bader charges were evaluated using all-electron charge densities^35,36^. Details on the specific calculations are given in the SI^37–46^.

The catalytic studies for propane dehydrogenation were performed in a continuous flow laboratory setup. The IMC powders were weighed (202.3 mg of Ga_3_Rh and 150.0 mg of Ga_9_Rh_2_) and mixed with a silica support (~ 1.5 g). The IMC-support mixture was transferred into a tubular, fixed-bed quartz reactor (length: 650 mm; inner diameter: 10 mm). The catalytic testing was conducted at 550 °C. The catalyst was first pre-treated in a flow of 20% hydrogen in helium for 3 h. After purging the reactor with helium for 1 h, the catalytic activity of the IMCs was tested in a flow of 8.9 mL_N_ min^−1^ propane (99.95% purity, Linde Gas) as feed gas diluted in 89 mL_N_ min^−1^ helium (99.996% purity, Linde Gas).

Soft/Hard x-ray photoelectron spectroscopy (XPS/HAXPES) experiments were conducted at the SISSY-1 endstation at the Energy Materials In-Situ Laboratory (EMIL) of Helmholtz-Zentrum-Berlin (HZB). The SISSY-1 setup houses a Scienta EW 4000 hemispherical electron analyzer that together with the two-color beamline of EMIL is capable to perform photoelectron spectroscopy in the soft and hard X-ray regime at UHV conditions (base pressure < 2∙10^−9^ mbar). The soft X-rays from 100 to 1500 eV are provided by the UE48 PGM undulator beamline. In addition, hard X-rays from 2000 to 10,000 eV are provided by the U17 DCM undulator beamline, which is also focused in the SISSY-1 end-station at the same position on the sample.

## Results and discussion

We prepared two polycrystalline intermetallic compounds of the stoichiometry Ga_9_Rh_2_ and Ga_3_Rh in a vacuum process (see SI), both of which are expected to show isolated, catalytically active surface sites. The compositional and structural character of the two compounds was investigated by energy dispersive X-ray spectroscopy (EDXS), high-resolution imaging, and electron diffraction tomography (EDT) within a transmission electron microscope (TEM) as well as powder X-ray diffraction (XRD) and structure refinement. For Ga_9_Rh_2_, the structure deduced from XRD and electron diffraction tomography (EDT) is in good agreement with the space group Pc, which is expected from the phase diagram^47^. HRTEM lattice imaging additionally revealed a high perfection of lattice stacking in thin regions of some crystallites of Ga_9_Rh_2_. (SI, Fig. S6).

**Figure 1:**
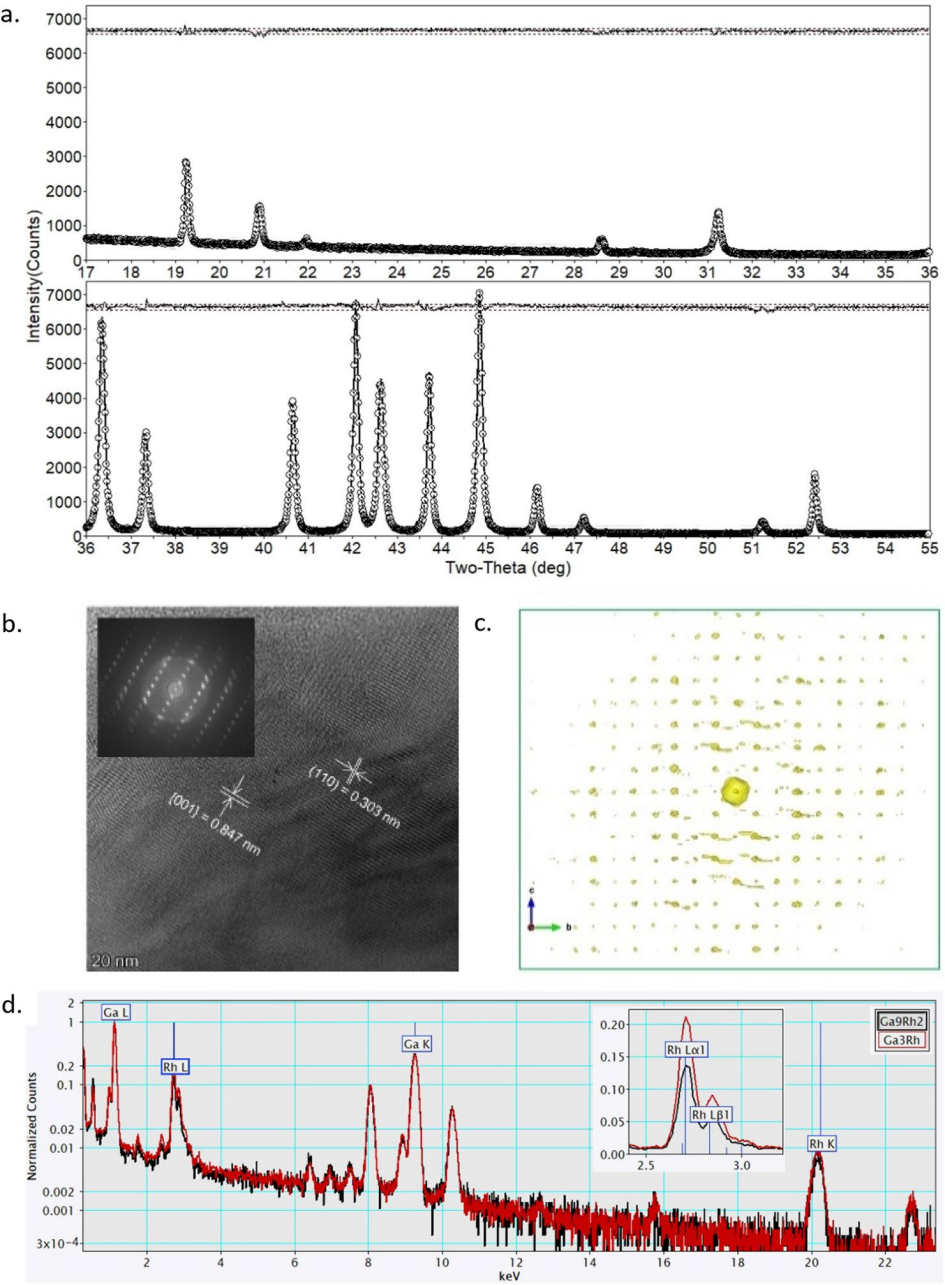
(**a**) XRD results of Ga_3_Rh and Rietveld refinement of Ga_3_Rh in space group Cmc2_1_ (for details see text); (**b**) HRTEM image of Ga_3_Rh from a thin region of a crystallite projected close to [− 110] direction. The image indicates tilting (i.e. waviness) of the (001) lattice planes; (**c**) 3D EDT data viewed along [100] direction (the [100] 2D slice does not contain enough information due to missing wedge and limited tilt range); (**d**) EDX spectra of crystallites of Ga_3_Rh and Ga_9_Rh_2_ from thin crystalline regions with negligible absorption effect. The quantitative results using the Rh-L/Ga-K signals agree well to their respectively expected stoichiometry.

For Ga_3_Rh, HRTEM, EDT, EDX and XRD analysis, Fig. 1, yield a new structure type with the space group (Cmc2_1_); this structure is in contrast to published data, which suggested that Ga_3_Rh is isostructural to IrIn_3_^19,20,48^.

Rietveld refinement indicates that the Ga_3_Rh crystallites are plate-like with the thin dimension in the [100] direction of the crystal lattice as deduced from a single line fit of three Bragg reflections 200 (57.235°), 150 (57.789°) and 025 (57.992°) with a Pearson VII profile, see SI Fig. S4a. The different FWHM (Full Width at Half Maximum) of the reflections show the directional anisotropy of the crystallite shape. The FWHMs are: 200 0.294(6) °2Θ, 150 0.108(3) °2Θ, and 025 0.155(4) °2Θ. The broad 200 reflection indicates the smaller width of the crystallites in the [100] direction. This plate-like size anisotropy resulted in a preferred orientation of the powder upon sample preparation. The fit indicates a mixed orientation with the main preferred orientation axes [001] and [011]. From HRTEM, disorder in the stacking of the edge-sharing plane of the GaRh antiprism cages was observed both in imaging and in many of the diffraction datasets (SI, Fig. S8). The elemental compositions of the two compounds as determined from EDXS data (taking into account absorption effects, cf. SI) almost perfectly matches the expected values with Rh concentrations of 17.8 (± 2.7) % (Ga_9_Rh_2_: 18.2%) and 25.2 (± 2.7)% (Ga_3_Rh:25%), respectively.

**Figure 2:**
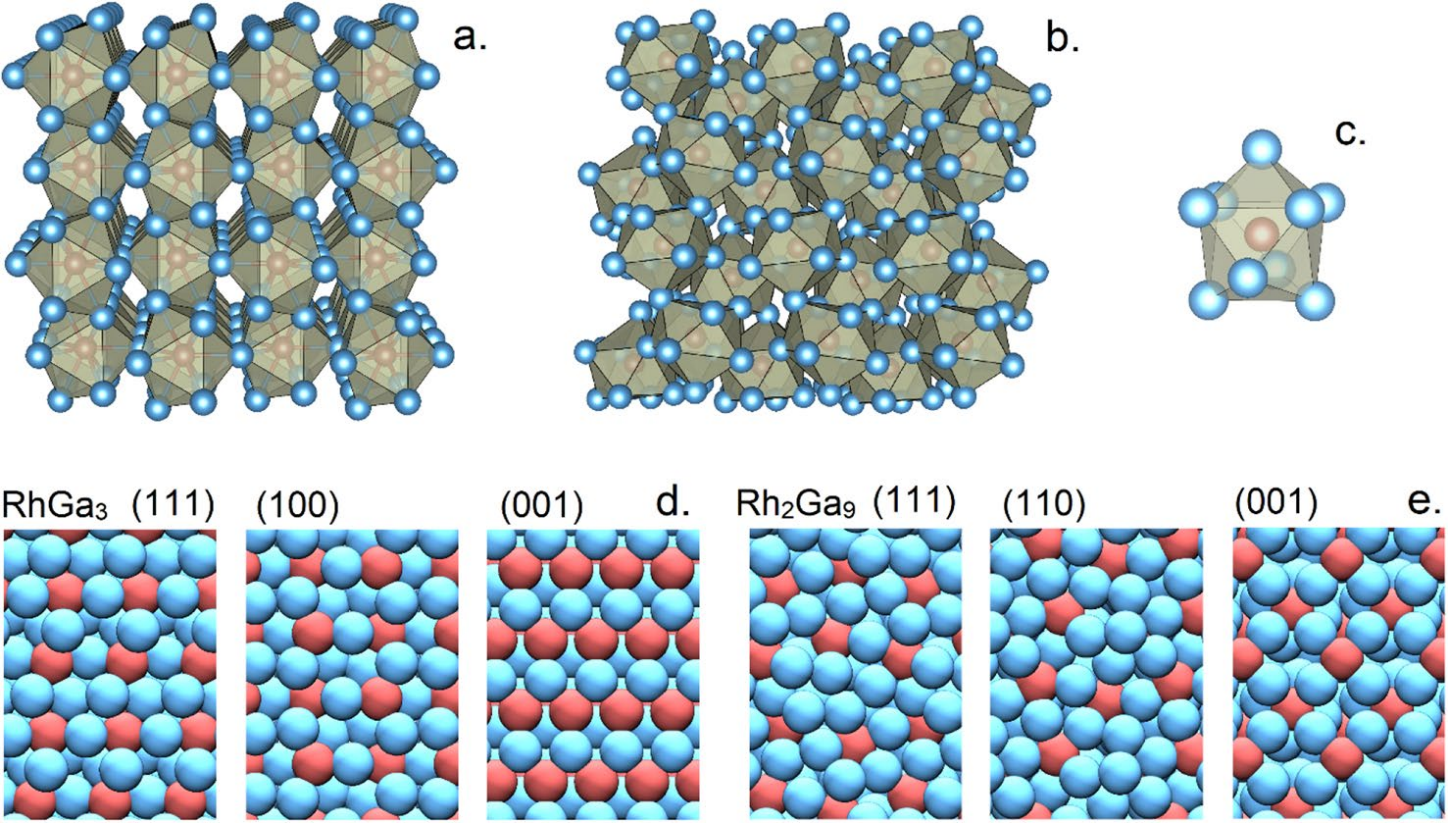
(**a**) Ga_3_Rh structure (**b**) Ga_9_Rh_2_ structure. Both structures are networks of Ga_9_Rh polyhedra; for comparison one polyhedron is shown in (**c**). In (**d**) and (**e**) the three most stable Ga_3_Rh and Ga_9_Rh_2_ surfaces identified by DFT are depicted. (Ga is shown in blue, Rh in red. The green polyhedra are a guide to the eye).

The building block in both Ga_3_Rh and Ga_9_Rh_2_ is a single-capped square antiprismatic coordination polyhedron around the metal atom Rh^17,18^. The two structures are shown in Fig. 2a and b, respectively, along with the structure of a single polyhedron in Fig. 2c (details on distances and bond angles are given in the SI in Tables S2, S3, and Fig. S5; Table S1 tabulates the refined structure data obtained from the Rietveld fitting of Ga_3_Rh). In Ga_3_Rh (Cmc2_1_), the polyhedra form sheets in the a-c planes of the orthorhombic structure. Within these sheets, the polyhedra share edges with one neighboring polyhedron. In b-direction, the sheets of polyhedra are interconnected by corners.

With the increase of the Ga:Rh ratio from 3:1 for Ga_3_Rh to 4.5:1 for Ga_9_Rh_2_ (Pc), the connectivity of the single-capped square antiprismatic coordination polyhedra increases. In Ga_3_Rh, sheets of polyhedra are connected by one corner on the left and one corner on the right. In Ga_9_Rh_2_, polyhedra share one edge with a single neighboring polyhedron but are connected to further neighboring polyhedra by seven corners. Together with the shared edge, this leads to a dense three-dimensional connectivity of the polyhedra network. The experimentally determined unit cells for Ga_3_Rh, and Ga_9_Rh_2_ (and also for a pure Rh crystal) are well reproduced by periodic density-functional theory (DFT) calculations using the PBE functional, with a slight enlargement of the unit cell of 2.5%. (Table S1, SI for Ga_3_Rh (Cmc2_1_), ref.^17,18^ for Ga_9_Rh_2_ (Pc) and Table S4, SI for the DFT data). The calculated formation energies per atom are − 0.47 eV for Ga_3_Rh and − 0.36 eV for Ga_9_Rh_2_.

For a more detailed insight into the surface morphology, we evaluated the surfaces energies of all low index surfaces (with a maximum Miller Index of 1) for different surface terminations using DFT slab calculations. A more thorough description is given in the SI. The three most stable low index surfaces of the Ga_3_Rh and Ga_9_Rh_2_ structures identified in the calculations are shown in Fig. 2d and e, respectively. For both structures, well isolated Rh sites are present that potentially are reactive and stable. The minimum distance between two Rh sites is ~ 4.7 Å in almost all cases, except for the Ga_3_Rh (001) surface with the smallest Rh-Rh distance of 3.3 Å, and thereby larger than a C–C bond distance of 1.5 Å and longer than e.g. a propylene molecule including the hydrogen atoms (~ 4.5 Å) that was used in the catalytic tests (see below).

**Figure 3:**
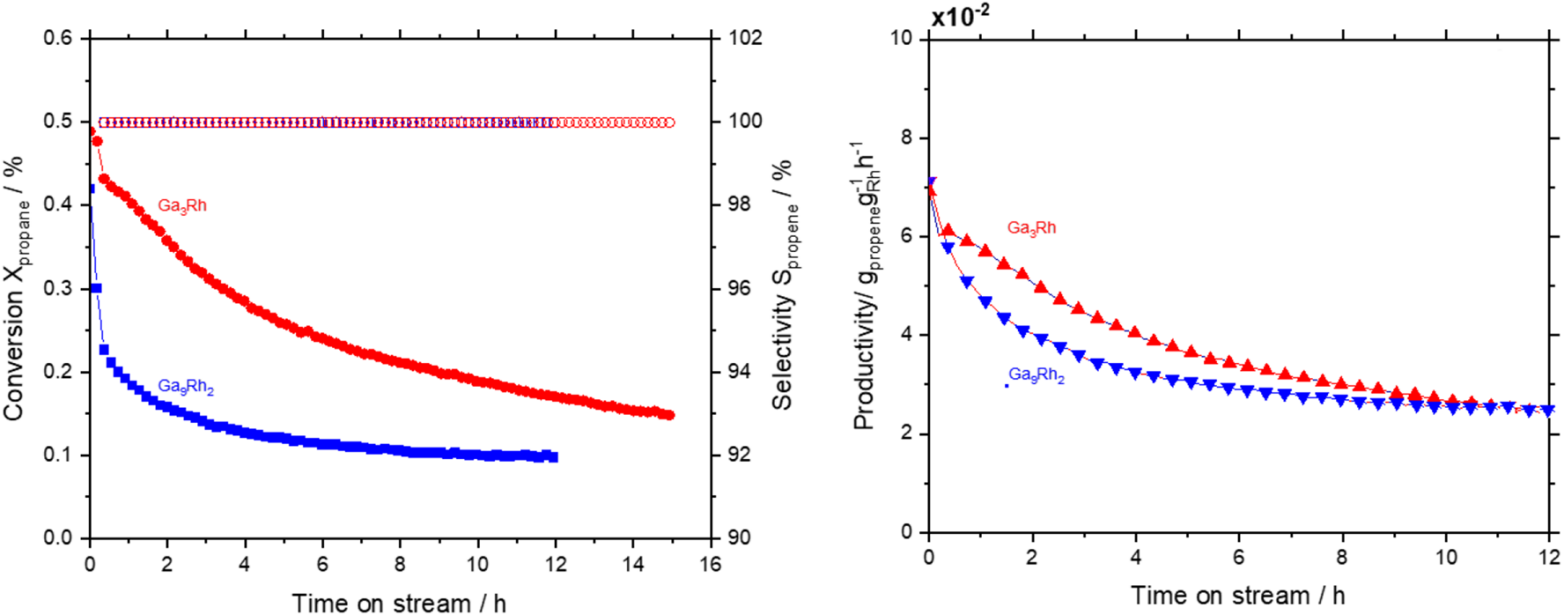
Continuous gas-phase propane dehydrogenation using GaRh IMC catalysts. For Ga_3_Rh (red symbols) and Ga_9_Rh_2_ (blue symbols) the conversion (filled) and selectivity (open) are shown over the time on stream in the right figure. The left figure shows productivities i.e. the mass of propene produced per mass of Rh. Reaction conditions: 550 °C, 1.2 bar, catalyst bed composition: 202.3 mg Ga_3_Rh + 1.5 g silica; 150.0 mg Ga_9_Rh_2_ + 1.5 g silica, Gas flows: He flow 89 mL_N_ min^–1^, C_3_H_8_ flow 8.9 mL_N_ min^–1^.

To test the hypothesis that these Ga intermetallic compounds (IMC) are active in dehydrogenation catalysis, we conducted catalytic tests for propane dehydrogenation (PDH) in a fixed bed tubular reactor. Silica with a particle size of 60–200 µm was chosen as support as it shows only minimal blind activity in PDH. XRD shows that no structural changes occurred during the catalytic tests, see SI. Both IMCs showed activity for propane dehydrogenation (see Fig. 3) with an initial conversion of 0.5% for Ga_3_Rh and 0.4% for Ga_9_Rh_2_, accompanied by a reduction in conversion (deactivation) with time on stream, a behavior that is known for classical heterogeneous catalyst as well as SCALMS^5^. This deactivation most likely originated from coke deposition on the active metal surface, as we observe an increased carbon content (see SI). At these low conversion levels, the selectivity was completely in favor of propene. From the selectivity and conversion data, a Rh-based productivity was calculated (for details, see SI). The initial productivity for both IMC catalysts was rather low with only 0.07 g_propene_g_Rh_^−1^ h^−1^ compared to the GaRh SCALMS systems, which ranged between 50 and 250 g_propene_g_Rh_^−1^ h^−1^. This large difference, by a factor of 10^4^, resembled the significantly different particle sizes: while Rh-SCALMS droplets were in the range of 400 nm, the two IMC catalysts were larger by a factor of 100. Assuming a surface reaction, this scales to a lower surface area of the IMC of 10^–4^. Hence, one can assume similar activity of the Rh sites in both SCALMS and IMC.

**Figure 4:**
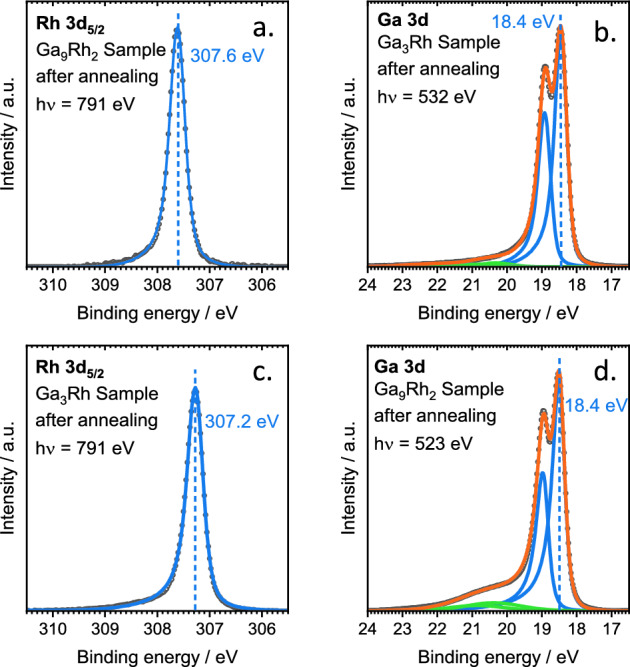
XPS spectra of the Rh 3d and Ga 3d spectroscopic regions for (**a,b**) Ga_9_Rh_2_ and (**c,d**) Ga_3_Rh.

To assess the surface and bulk composition and the electronic structure of both intermetallic compounds, they were investigated using synchrotron-based soft and hard X-ray photoelectron spectroscopy. The Rh 3d_5/2_ and Ga 3d, regions are shown in Fig. 4. For the Ga_3_Rh sample, the quantitative analysis of the XP spectra yields ~ 23 at.% Rh, which agrees well with the stoichiometry (25%). For Ga_9_Rh_2_, however, we observe only ~ 7 at.% Rh; this value is significantly smaller than the stoichiometry (18%), possibly due to overstochiometric amounts of Ga in the preparation that accumulate at the surface.

The XP spectra of both intermetallic compounds, Ga_9_Rh_2_ (Fig. 4b) and Ga_3_Rh (Fig. 4d) show mostly metallic Ga, with the spin–orbit split Ga 3d_5/2_ and 3d_3/2_ signals at 18.4 and 18.9 eV. The small peaks at 20.3 (20.8) eV (green curves) indicate minor amounts of Ga suboxides with 10% and 4% of the total intensity for Ga_9_Rh_2_ and Ga_3_Rh, respectively. The Rh 3d_5/2_ spectra in Fig. 3a and c show the main components at 307.6 eV for Ga_9_Rh_2_ and at 307.2 eV for Ga_3_Rh. This compares well to the calculated core level shifts in the final state approximation (Table S5, SI) relative to pure Rh (Ga_3_Rh: − 0.2 eV (exp.) and -0.12 eV (DFT); Ga_9_Rh_2_: + 0.2 eV (exp.) and + 0.22 eV (DFT) relative to pure Rh). A more detailed discussion of the core level shifts is given in the SI.

**Figure 5:**
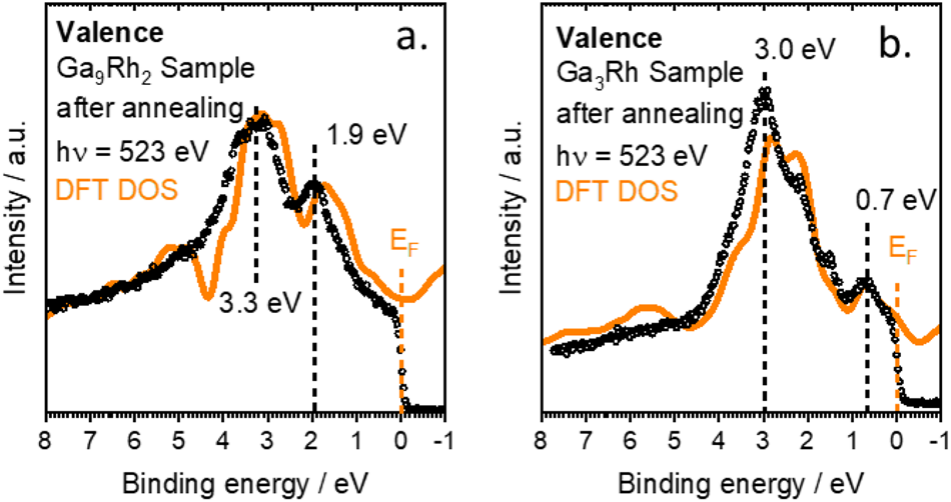
Combined valence band measurements (black dots) and calculated density of states (orange lines) for (**a**) Ga_9_Rh_2_ and for (**b**) Ga_3_Rh.

This behavior can be explained by the experimental and calculated DOS of the alloys shown in Fig. 5 for Ga_3_Rh, and Ga_9_Rh_2_. The total and partial electronic density of states (DOS) is shown in Fig. S14 for Rh, Ga_3_Rh, and Ga_9_Rh_2_. In the case of pure metallic Rh, a broad band is identified, which crosses the Fermi level due to the incomplete filling of the Rh 4d states. The IMCs exhibit broad Ga sp bands, which interact with the Rh states leading to a shift of the Rh states to larger binding energies, therefore decreasing the d-character of the states at the Fermi level.

At this point, the question arises, what is the influence of the geometric vs the electronic structure? While Rhodium is known to be a very poor PDH catalyst, mainly due to its strong tendency for coking, the implementation of Rh in a Ga matrix mitigates coking and the catalytic activity remains high over several hours. This improved catalytic activity is attributed to the site isolation that is found for the intermetallic compounds Ga_3_Rh and Ga_9_Rh_2_. The behavior is similar to what is observed for other single-atom catalysts, such as GaRh SCALMS. As discussed above, the site isolation is accompanied by a change in the electronic structure of the catalytically active Rh atoms: The position of the Rh d-band center moves away from the Fermi edge as a function of the Rh concentration in the Ga matrix and the negative charge centered at the Rh atom. Additionally, the width of the Rh d-band narrows. While these electronic effects have an influence on catalysis, the site isolation is expected to be the main influence for Rh to be active in PDH. As coking is the main issue in deactivation, the inability to accommodate carbon on the inactive Ga matrix is proposed to be the main driver to obtain catalytically active Rh single site atoms.

## Conclusion

Two intermetallic compounds with isolated reaction sites were prepared and characterized. For both Ga_9_Rh_2_ and Ga_3_Rh, we used XRD and TEM for structural characterization, while XPS and EDX were used to access the chemical composition. With DFT and XPS the electronic structure of the compounds was explored. In the structural analysis, Ga_9_Rh_2_ was identified based on its X-ray diffraction reference, while the Ga_3_Rh structure was solved and refined showing a new structure with orthorhombic space group Cmc2_1_. Both compounds exhibit isolated reaction sites at the surface that are active in propane dehydrogenation, while showing only minor deactivation. The insights into the electronic structure revealed that the Rh d band shifts significantly to larger binding energies and changes its shape. These results give insight into the effect of site isolation in catalysis, allowing for the utilization of new materials in catalysis, even under harsh conditions, such as in the case of the highly relevant dehydrogenation reactions.

## Supplementary Information

Supplementary Information.

## Data Availability

The crystal structure is available at https://www.crystallography.net/cod/3000399.html.
