# Author Correction: Isolated Rh atoms in dehydrogenation catalysis

**DOI:** 10.1038/s41598-023-35778-1

**Published:** 2023-06-13

**Authors:** Haiko Wittkämper, Rainer Hock, Matthias Weißer, Johannes Dallmann, Carola Vogel, Narayanan Raman, Nicola Taccardi, Marco Haumann, Peter Wasserscheid, Tzung-En Hsieh, Sven Maisel, Michael Moritz, Christoph Wichmann, Johannes Frisch, Mihaela Gorgoi, Regan G. Wilks, Marcus Bär, Mingjian Wu, Erdmann Spiecker, Andreas Görling, Tobias Unruh, Hans-Peter Steinrück, Christian Papp

**Affiliations:** 1 Lehrstuhl für Physikalische Chemie II, Friedrich-Alexander-Universität Erlangen-Nürnberg (FAU), Egerlandstr. 3, 91058 Erlangen, Germany; 2 Lehrstuhl für Kristallographie und Strukturphysik, Friedrich-Alexander-Universität Erlangen-Nürnberg (FAU), Staudtstr. 3, 91058 Erlangen, Germany; 3 Lehrstuhl für Chemische Reaktionstechnik (CRT), Friedrich-Alexander-Universität Erlangen-Nürnberg (FAU), Egerlandstr. 3, 91058 Erlangen, Germany; 4 Forschungszentrum Jülich GmbH, Helmholtz-Institute Erlangen-Nürnberg for Renewable Energy (IEK-11), Egerlandstr. 3, 91058 Erlangen, Germany; 5 Department Interface Design, Helmholtz-Zentrum Berlin für Materialien und Energie GmbH (HZB), 12489 Berlin, Germany; 6 Energy Materials In-Situ Laboratory Berlin (EMIL), HZB, 12489 Berlin, Germany; 7 Lehrstuhl für Theoretische Chemie, Friedrich-Alexander-Universität Erlangen-Nürnberg (FAU), Egerlandstr. 3, 91058 Erlangen, Germany; 8 Department X-Ray Spectroscopy at Interfaces of Thin Films, Helmholtz Institute for Renewable Energy (HI ERN), 12489 Berlin, Germany; 9 Lehrstuhl für Werkstoffwissenschaften (Mikro- und Nanostrukturforschung), Cauerstraße 3, 91058 Erlangen, Germany; 10 Physikalische und Theoretische Chemie, Freie Universität Berlin, Arnimallee 22, 14195 Berlin, Germany

Correction to: *Scientific Reports* https://doi.org/10.1038/s41598-023-31157-y, published online 17 March 2023

The original version of this Article contained an error in the spelling of the author Nicola Taccardi, which was incorrectly given as Nicola Tacardi.

The original Article has been corrected.

