# Supplementary material for: Isolated Rh atoms in dehydrogenation catalysis
Source: Sci Rep. 2023 Mar 17;13:4458. doi: 10.1038/s41598-023-31157-y (PMC10023779; doi:10.1038/s41598-023-31157-y)
Supplement: Supplementary file 1 — Supplementary Information. [file 41598_2023_31157_MOESM1_ESM.pdf]

Supporting Information:

Isolated Rh atoms in dehydrogenation catalysis

Haiko Wittkämper[1], Rainer Hock[2], Matthias Weißer[2], Johannes Dallmann[2], Carola Vogel[2], Narayanan Raman[3], Nicola Tacardi[3], Marco Haumann[3], Peter Wasserscheid[3,4], Tzung-En Hsieh[5,6], Sven Maisel[7], Michael Moritz[1], Christoph Wichmann[1], Johannes Frisch[5,6], Mihaela Gorgoi[5,6], Regan G. Wilks[5,6], Marcus Bär[1,5,6,8] Mingjian Wu[9], Erdmann Spiecker[9], Andreas Görling[7], Tobias Unruh[2], Hans-Peter Steinrück[1], Christian Papp[1,10]

1: Friedrich-Alexander-Universität Erlangen-Nürnberg (FAU), Lehrstuhl für Physikalische Chemie II, Egerlandstr. 3, 91058 Erlangen, Germany

2: Friedrich-Alexander-Universität Erlangen-Nürnberg (FAU), Lehrstuhl für Kristallographie und Strukturphysik, Staudtstr. 3, 91058 Erlangen, Germany

3: Friedrich-Alexander-Universität Erlangen-Nürnberg (FAU), Lehrstuhl für Chemische Reaktionstechnik (CRT), Egerlandstr. 3, 91058 Erlangen, Germany

4: Forschungszentrum Jülich GmbH, Helmholtz-Institute Erlangen-Nürnberg for Renewable Energy (IEK-11), Egerlandstr. 3, 91058 Erlangen, Germany

5: Department Interface Design, Helmholtz-Zentrum Berlin für Materialien und Energie GmbH (HZB), 12489 Berlin, Germany

6: Energy Materials In-situ Laboratory Berlin (EMIL), HZB, 12489 Berlin, Germany

7: Friedrich-Alexander-Universität Erlangen-Nürnberg (FAU), Lehrstuhl für Theoretische Chemie, Egerlandstr. 3, 91058 Erlangen, Germany

8: Department X-ray Spectroscopy at Interfaces of Thin Films, Helmholtz Institute for Renewable Energy (HI ERN), 12489 Berlin, Germany

9: Lehrstuhl für Werkstoffwissenschaften (Mikro- und Nanostrukturforschung), Cauerstraße 3, 91058 Erlangen, Germany

10: Physikalische und Theoretische Chemie, Freie Universität Berlin, Arnimallee 22, 14195 Berlin, Germany

Shared corresponding authors: Matthias Weißer (matthias.weisser@fau.de), Christian Papp (christian.papp@fau.de)

Sample preparation and visual inspection:

Synthesis of Ga_9Rh_2 and Ga_3Rh

We synthesized Ga_9Rh_2 and Ga_3Rh, the first two intermetallic phases on the Ga-rich side of the partially known RhGa phase diagram. For the synthesis, we weighed in stoichiometric amounts of Rh wire (99.9 % Goodfellow) and liquid Ga (99.99999 % Sigma-Aldrich) into Al_2O_3 crucibles (Almath 13 mm x 10.5 mm). Weigh-in was done in air. To avoid excessive oxidation induced by heating, synthesis was performed under vacuum conditions (< 10^{-5} mbar) in a 5.6 kW resistance heated (graphite) vacuum furnace, designed for temperatures up to 1800°C, see Fig. S1. To ensure full mixing of both components and to be above the melting temperature of the intermetallic compounds, we aimed for a temperature of 1000°C. According to the known phase diagram, Ga_9Rh_2 is expected to be fully liquid at around 740°C and Ga_3Rh at around 950°C. After pumping the oven down to 10^{-5} mbar, the samples were slowly heated to 1000°C over the next 5 hours. The samples were kept at 1000°C for 10 min and afterward cooled down to 500°C at a cooling rate of 0.8°C per minute. After reaching 500°C, the samples were kept at this temperature for up to 10 h before cooling to room temperature.

For both compositions, the synthesis resulted in small grayish ingots that visibly differ from pure Ga or Rh. Visual inspection and light microscopy already suggest that both samples are polycrystalline with Ga_9Rh_2 being more powdery, with smaller crystalline domains, see Fig. S2. For Ga_3Rh, the visible crystal facets are significantly larger with ~0.15 mm edge length as compared to Ga_9Rh_2 with ~0.03 mm edge length.

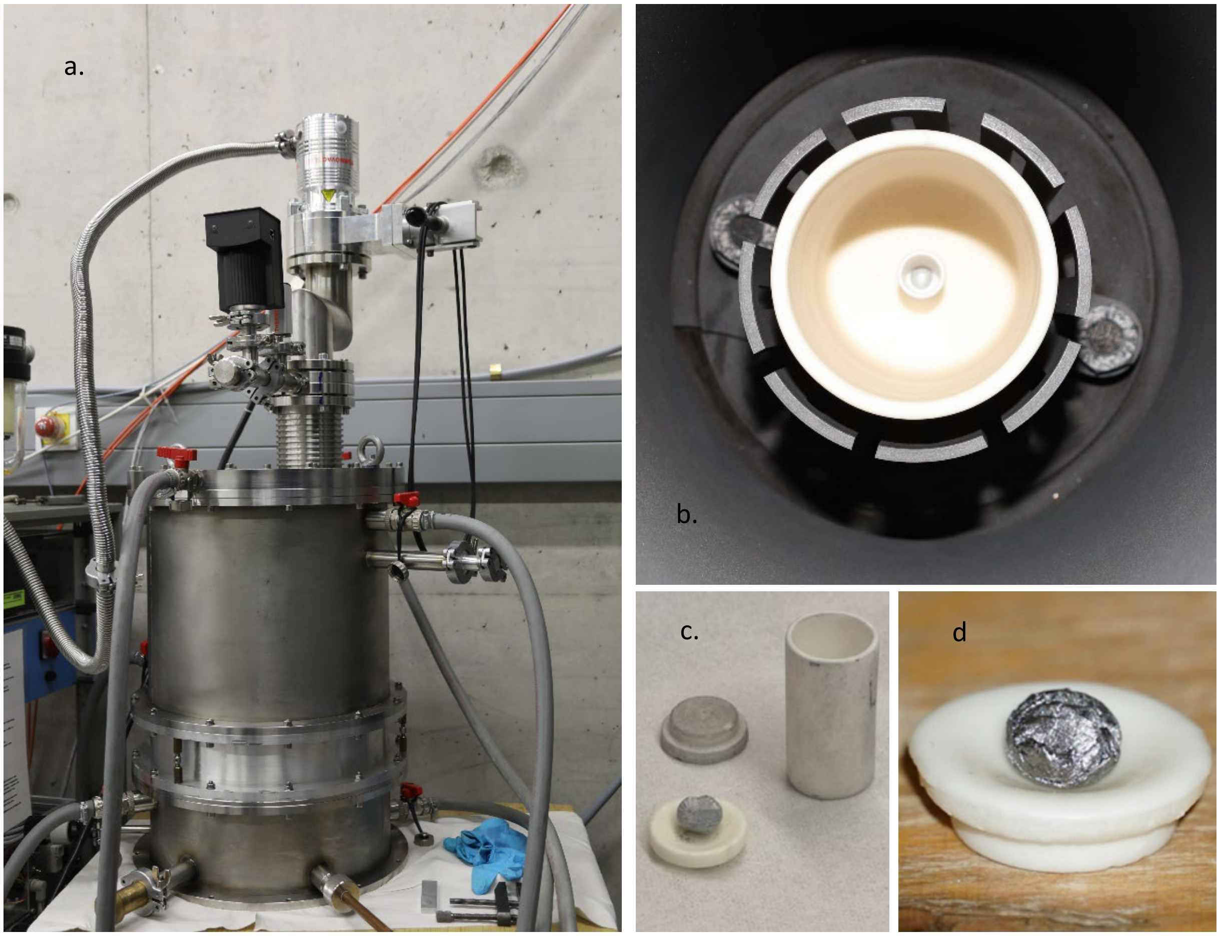

Figure S1: a, b) Photographs of the vacuum oven; alumina crucible with c) a Ga_9Rh_2 ingot and d) a Ga_3Rh ingot.

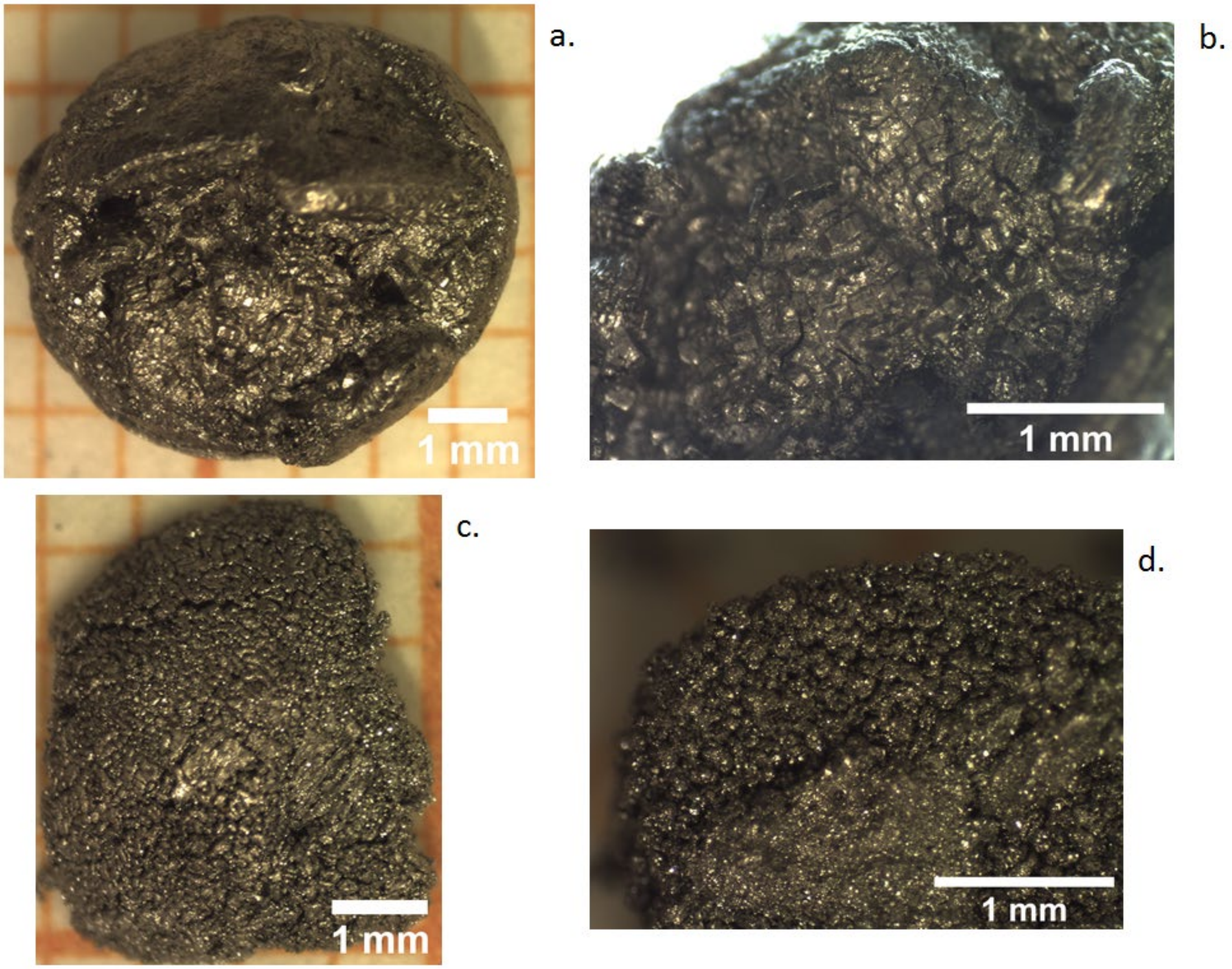

Figure S2: Magnified photographs of the obtained ingots, a, b) Ga_3Rh and c, d) Ga_9Rh_2.

X-ray Diffraction and Structure Refinement:

Characterization

Both synthesized alloys were crushed in an agate mortar to produce a fine powder.

Ga_9Rh_2 was measured with a Panalytical X'pert powder diffractometer and Ni filtered Cu $K\alpha_{1,2}$ radiation in Θ–Θ geometry. The diffraction pattern was recorded in an angular range from 15° – 120° 2Θ by an XCelerator detector module in 1D mode with a step size of 0.00836° and a counting time of 500s per step. The synthesis of Ga_9Rh_2 was verified by a Rietveld refinement based on the published structure data by Boström et al. [1] to the measured powder pattern. The structure was refined with the program JADE 9 in space group Pc with the published lattice parameters, the atomic coordinates and the individual isotropic temperature factors (ICSD 414305) as starting values. The isotropic temperature factors were kept fixed in the refinement. The agreement between the published structure and the result of the Rietveld refinement of the synthesized Ga_9Rh_2 is good, see Fig. S3. Some stronger deviations between calculated and measured diffraction intensities are due to the graininess of the powder. The particle size distribution is broad and includes crystallites with a size exceeding the optimal size (>10µm) for a powder diffraction experiment with good powder statistics. The refined lattice parameters and atomic coordinates nevertheless compare well with the published data.

The powder pattern of Ga_3Rh was measured in Θ–Θ geometry with a Rigaku SmartLab copper target rotating anode. The excitation voltage was 45 kV and the anode current was 160 mA. The primary beam was monochromatized by a Ge Johansson monochromator. The diffracted signal was detected by a Rigaku HyPix-3000 detector used in 1D mode. The measurement was performed with a continuous measurement speed of 0.4°/min and angular step width of 0.01 °2Θ. The measured angular range of the powder diagram is 15° - 100° 2Θ. The peak to background ratio based on the intensity of the most intense Bragg reflection, observed at about 44.9 °2Θ, is 1:60. We compared our measured diffraction pattern of Ga_3Rh to calculated diffraction patterns of In_3Ir based on previously published data for the two space groups.[2, 3] Indium was replaced by Ga and Iridium by Rh in our simulations. Powder patterns of In_3Ir and the diffraction pattern observed on our Ga_3Rh sample are substantially different. They cannot be confused, even so, one must certainly expect and account for different lattice parameters of In_3Ir and a hypothetical Ga_3Rh structure

isotypic to In_3Ir. This non-agreement of the diffraction patterns is expected from the different space groups of the crystals and the different extinction rules. We conclude that Ga_3Rh crystallized in space group $Cmc2_1$ and thus is a new structure type in the group of Ga-Rh alloys.

Structure Solution & Structure Refinement for Ga_3Rh

Based on the measured powder diffraction pattern, the structure of Ga_3Rh was solved with the program JADE 9 and the structure solution program RUBY.[4] Indexation and subsequent Pawley fitting were done with JADE 9. In a first step, the Bragg angles of 27 well resolved Bragg reflections up to 70° 2Θ were fitted by single peak fitting with a Pearson VII profile function. The fitted reflection positions were used for indexation, unit cell search, and lattice parameter refinement as well as for a space group proposal. The best match was obtained for the space group $Cmc2_1$.[5] Lattice parameters after refinement including a zero point error were a = 3.2106(5) Å, b = 9.1660(10) Å, and c = 8.4571(8) Å for the orthorhombic metric. The first refined unit cell volume is V = 248.88 Å^3. With this choice of space group and starting lattice parameters, the entire diffraction pattern was fitted by a structureless Pawley Fit. The fit resulted in slightly modified lattice parameters a = 3.21170(4) Å, b = 9.16675(1) Å, and c = 8.45838(9) Å and a unit cell volume V = 249.02 Å^3. All observed reflections were well explained by the fit in space group $Cmc2_1$. The final Bragg R-factor of the structureless fit was about 6 %. To obtain this final best fit, individual reflection widths had to be included in the fitting procedure, indicating peak width anisotropy, an example is given in Fig. S4a. The indexed reflection intensities extracted by the Pawley Fit were transferred to the structure solution program RUBY. As the atomic unit cell content four formula units Ga_3Rh ($Ga_{12}Rh_4$) were chosen. For the refined unit cell volume, this atomic content results in a reasonable density of 8.324 g cm^{-3} for the alloy and is in accordance with the composition of the synthesis. The structure was then solved by direct methods. All atoms, three generating Ga atoms and one Rhodium atom, occupy Wyckoff positions 4a (0,y,z) in space group $Cmc2_1$. The atom positions of Ga and Rh taken from the direct methods solution were used as starting values in the subsequent Rietveld refinement.

To validate the solution found by direct methods, we solved the structure independently in real space with the program ENDEAVOUR.[6, 7] The program is made for structure solution from powder diffraction data. It combines a global optimization of the difference between calculated and measured diffraction patterns and uses the potential energy of the system by "Pareto

Optimization" and Simulated Annealing. In short, the atom positions in the unit cell of the known metric are varied, until the calculated intensities of the diffraction pattern are in best accordance with the measured diffraction intensities. As input for ENDEAVOUR, we used the lattice parameters obtained from the Pawley fit and the integrated intensities of 23 non-overlapping Bragg reflections up to 60.5° 2Θ. Four formula units (Z = 4) of Ga_3Rh were given to the program as unit cell content. Two structure solution attempts were followed, the solution in space group $Cmc2_1$ and a solution in space group P1. In P1 no symmetry restrictions are used in the solution process. ENDEAVOUR was run in default mode. All parameters for the structure solution were taken automatically pre-defined in the program. Both solution strategies performed well comparable results for the atom positions within the unit cell. And both solutions are in good agreement with the structure model obtained by direct methods with RUBY.

Starting from the structure solution output from RUBY, the Ga_3Rh crystal structure was refined with the Rietveld program WPF (**W**hole **P**attern **F**itting), a part of the program JADE 9. The fit was repeated with the Rietveld program MAUD (**M**aterials **A**nalysis **U**sing **D**iffraction).[8] Within the error, both programs resulted in similar fit results. To fit the powder diagram, in both Rietveld programs an anisotropic reflection width had to be fitted and the refinement of a preferred orientation was necessary. The fitted value of B corresponds to an average atomic vibration amplitude of <u> = 0.17 Å. A plot of the final Rietveld refinement of the structure model in space group $Cmc2_1$ to the measured data is given in the main text Fig. 1a. The single line fit of three Bragg reflections 200 (57.235°), 150 (57.789°), and 025 (57.992°) with a Pearson VII profile function is shown in Fig. S4a, showing the anisotropy in the diffraction.

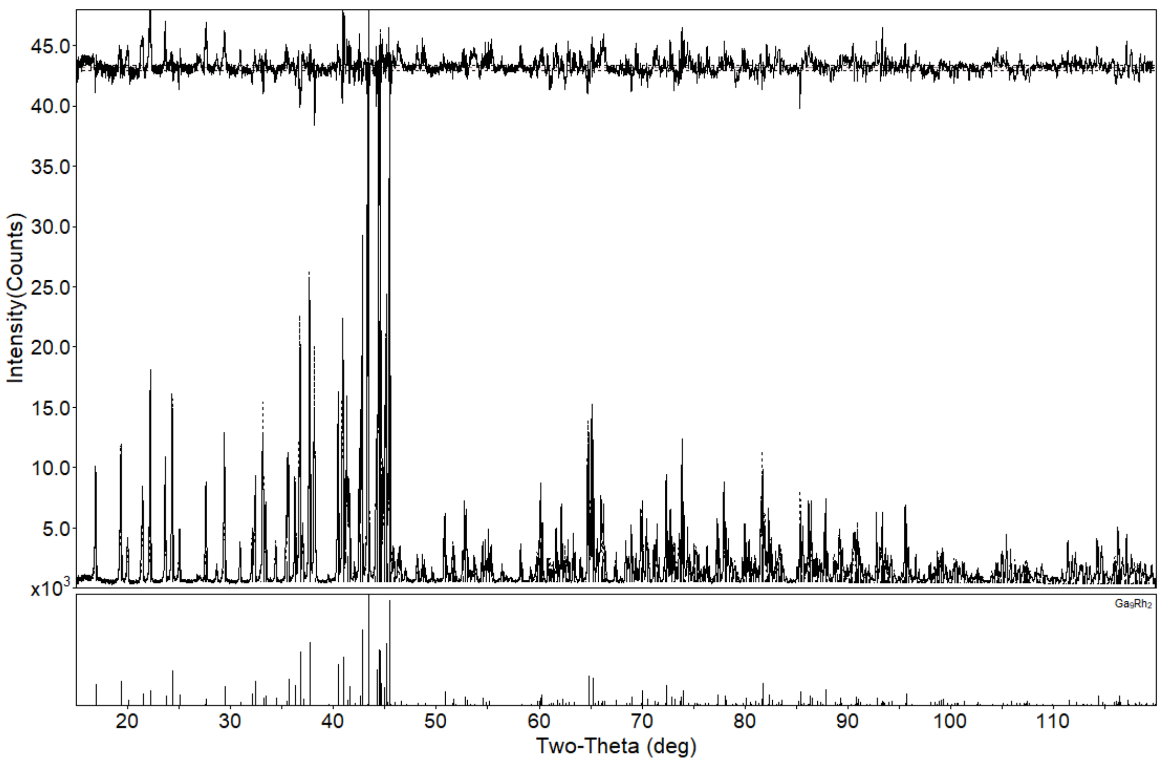

Figure S3: Rietveld refinement of Ga_9Rh_2 in space group Pc with the program WPF (Whole Pattern Fitting module in JADE 9). Above the fitted powder pattern, the difference plot between measured and calculated intensities is shown. Refined monoclinic lattice parameters are a = 6.4164(4) [6.4164(4)], b = 6.4126(4) [6.4119(4)], c = 8.7855(4) [8.7847(4)], β = 93.398(1) [93.390(5)]. Values in parenthesis are the lattice parameters published by Boström et al. [1].

a)

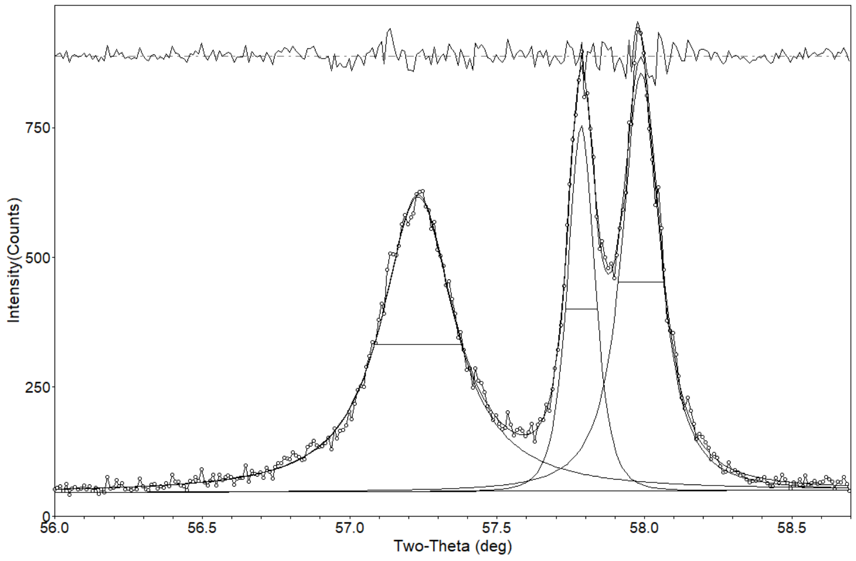

Figure S4: XRD of Ga_3Rh **a)** Single line fit of three Bragg reflections 200 (57.235°), 150 (57.789°), and 025 (57.992°) with a Pearson VII profile function. The different FWHM (Full Width at Half Maximum) of the reflections show the directional anisotropy in crystallite size. The FWHM are: 200 0.294(6) °2Θ, 150 0.108(3) and 025 0.155(4) °2Θ. The broadest 200 reflection indicates the smallest width of the crystallites in the [100] direction. The indexation is based on the determined space group $Cmc2_1$ of Ga_3Rh.

Table S1 shows the crystal structure data of Ga_3Rh obtained from the Rietveld fit in space group $Cmc2_1$. In Table S2, we report selected bond distances calculated from the refined atomic coordinates, as determined with WPF (Whole Pattern Fitting module in JADE 9). Bond distances are given within a polyhedron with Rh as the central atom and three long Ga-Ga distances which are equal to the length of the crystallographic a-axis of the unit cell. In Table S3 nine selected bond angles Ga_i – Rh1 – Ga_j (i, j = 1,2,3; only for $i \neq j$) with Rh as central atom are given. Fig. S5 shows a $RhGa_9$ coordination polyhedron with the corresponding bond distances.

Table S1: Crystal structure data of Ga_3Rh obtained from the Rietveld fit in space group $Cmc2_1$ (No. 36 International Tables for Crystallography) with the program WPF.

Crystal structure of Ga_3Rh	
Chemical formula	$Ga_{12}Rh_4$
Space Group	$Cmc2_1$, orthorhombic (No. 36)
Lattice parameters	
	a = 3.2116(1) Å
	b = 9.1666(1) Å
	c = 8.4581(1) Å
	$\alpha = \beta = \gamma = 90°$
Unit cell volume	V = 249.00(3) $Å^3$
Z	4
Calculated density	ρ = 8.324(1) g cm^{-3}
Chemical element, Wyckoff position, and fractional atomic coordinates	
Rh1	4a: 0.0 0.2357(1) 0.364(9)
Ga1	4a: 0.0 0.636(1) 0.581(8)
Ga2	4a: 0.0 0.9507(2) 0.358(8)
Ga3	4a: 0.0 0.6329(1) 0.159(8)
Overall isotropic B-factor	B = 2.34(2) $Å^2$
Average atomic vibration amplitude	<u> = 0.17 Å
Criteria for the goodness of fit	
Weighted Bragg R-value	R_{Bragg} = 7.66%
Expected E value	E = 7.06%
Ratio R/E	R/E = 1.1

Table S2: Selected bond distances for Ga_3Rh between Ga-Rh and Ga-Ga within the polyhedron and three long Ga-Ga distances equal to the length of the unit cell a-axis.

Rh-Ga bond distances in polyhedron	
Atom pairs	Bond distance [Å]
Rh1 – Ga1 (two bonds)	2.6208
(1 bond)	2.6875
Rh1 – Ga2 (2 bonds)	2.5427
(1 bonds)	2.6130
Rh1 – Ga3 (2 bonds)	2.5442
(1 bond)	2.7707
Closest Ga-Ga bond distances	
Ga1 – Ga2	2.9375
Ga1 – Ga2	2.9779
Ga1 – Ga3	2.5074
Ga1 – Ga3	2.7745
Ga2 – Ga3	2.8637
Ga2 – Ga3	3.1060
Long Ga-Ga distances	Equal to a-axis length
Ga1 – Ga1	3.2116
Ga2 – Ga2	3.2116
Ga3 – Ga3	3.2116

Table S3: Ga_3Rh: Nine selected bond angles Ga_i – Rh1 – Ga_j (i, j = 1,2,3; only for i ≠ j) with Rh as the central atom

Ga_i – Rh1 --Ga_j bond angles in polyhedron	
Atoms	Bond angle [°]
Ga1 - Rh1 – Ga2	69.36
	84.89
	133.22
Ga1 - Rh1 – Ga3	61.87
	87.42
	136.75
Ga2 - Rh1 – Ga3	71.40
	82.81
	132.23

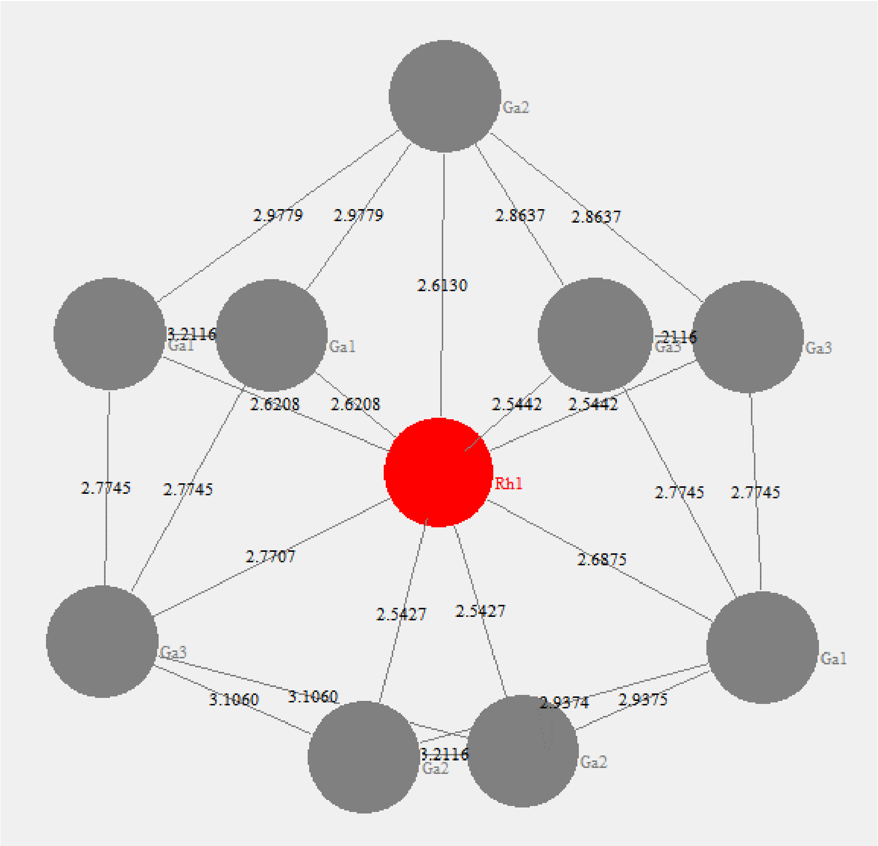

Figure S5: Single-capped square antiprismatic coordination polyhedron around the metal atom Rh and bond distances within the polyhedron.

TEM and Electron diffraction tomography

The crushed sample powder was supported on a standard 200 mesh Cu TEM grids filled with holey carbon network. The TEM grids were then mounted in a Fishione single tilt tomography holder and studied using a ThermoFischer Scientific Titan Themis monochromated, double Cs-corrected TEM.

The composition of the crystallites is evaluated with energy-dispersive X-ray spectrometry (EDXS) with data collected by a Super-X detector array equipped on the TEM. The dispersion was set so to cover an energy range up to 40 keV such that Rh-K, Rh-L, Ga-K and Ga-L family is recorded with sufficient resolution. The relative intensity ratio of Ga-L/Ga-K and Rh-L/Rh-K should be constant if absorption is negligible. These ratios thus allow inspection of the absorption effect. We noticed that the effective absorption length of emitted X-rays as probed in TEM-EDX mode (illumination of few micrometers covering the entire crystallites) heavily depends on the exact geometry of each crystallite and is difficult to disentangle. This makes quantitative comparison between different samples unreliable. For quantitative comparison, data with minimum (negligible) absorption effect is desirable. Thus, the EDX data were excited using a defocused electron probe in STEM mode (~10-20 nm probe size) on thin regions at the edge of crystallites and the EDX signals were collected using detector segments that are directly facing the edge of crystallites. Signals from segments showing shadowing or absorption are disregarded. The acquired data are evaluated using the Velox software with a standard k-factor method. The results reported are quantified based on the Ga-K and Rh-L family using the well accepted Braon-Powell ionization cross-section model as implemented in the Velox software.

Well separated single crystalline particles of 500 - 2000 nm in size were selected for electron diffraction tomography (EDT) study. EDT data were collected typically from -70 to 70 degrees in continuous tilting while the camera was capturing at 10fps. Typical datasets are 350 – 600 frames. The unit cell parameters, 3D reciprocal lattice as well as the virtual 2D diffraction slices are processed using the PETS software. These single crystal diffraction patterns are compared with simulated diffraction patterns from the literature structure (in case of Ga_9Rh_2) and the structure determined via powder XRD (in case of Ga_3Rh) as validation.

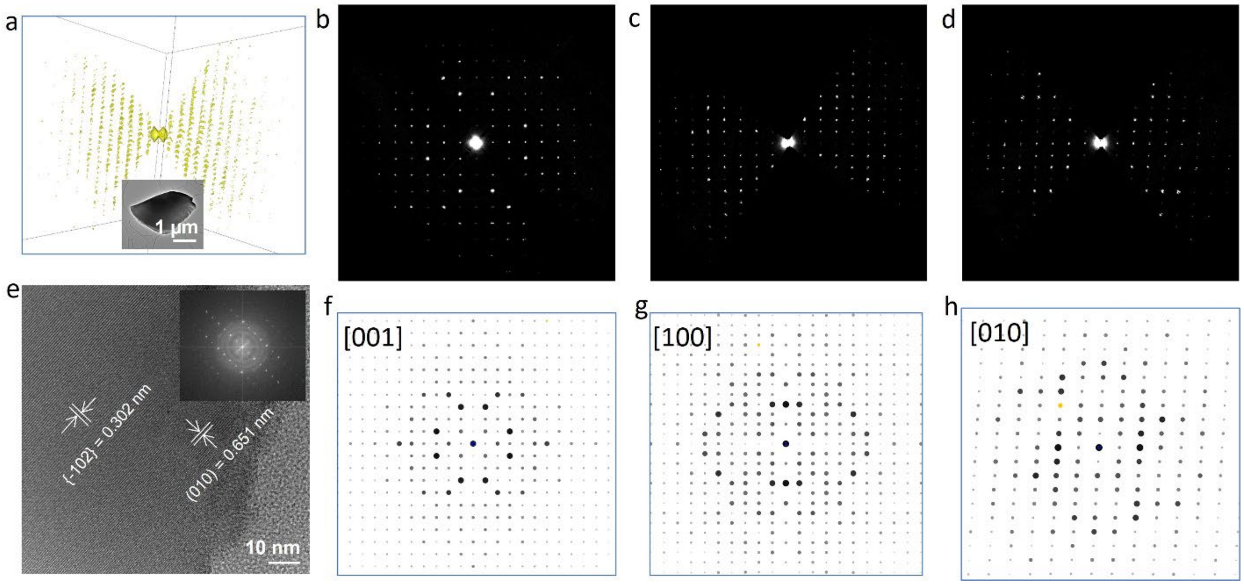

Figure S6. TEM analysis of Ga_9Rh_2 single crystalline phase. (a) 3D view of the electron diffraction tomography data. Inset shows the micro-crystallite from which the diffraction data were collected. (b), (c) and (d) are the 2D slices of the 3D data along the [001], [100], and [010] direction, and the respectively simulated 2D pattern using the literature structure (ICSD No. 635207) are shown in (f) – (h). (e) a HRTEM image (and Fourier transform of the image as inset) of a thin region from another crystallite close to the [201] zone axis.

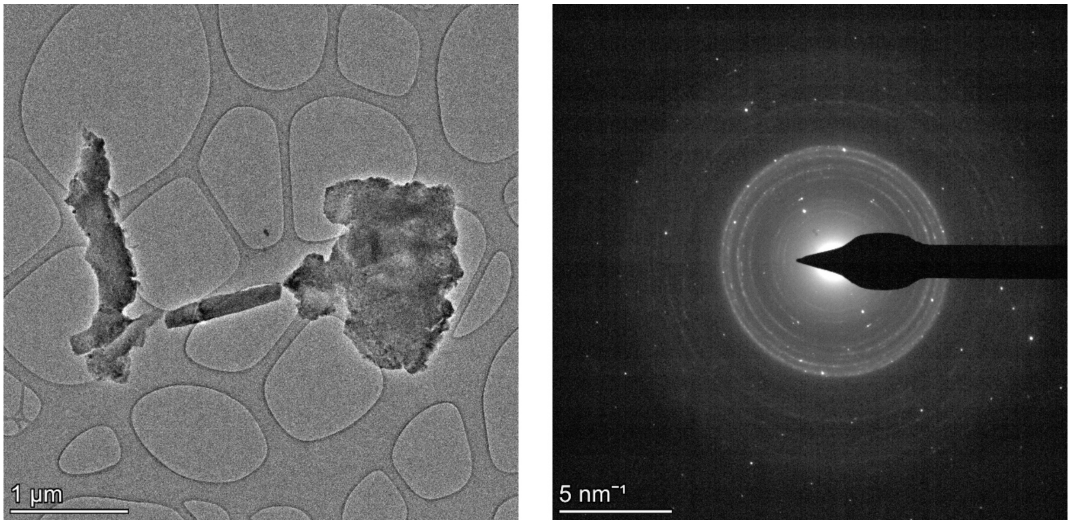

Figure S7. TEM image (left) and selected area electron diffraction (right) of aggregates of nano-crystallites of Ga_9Rh_2.

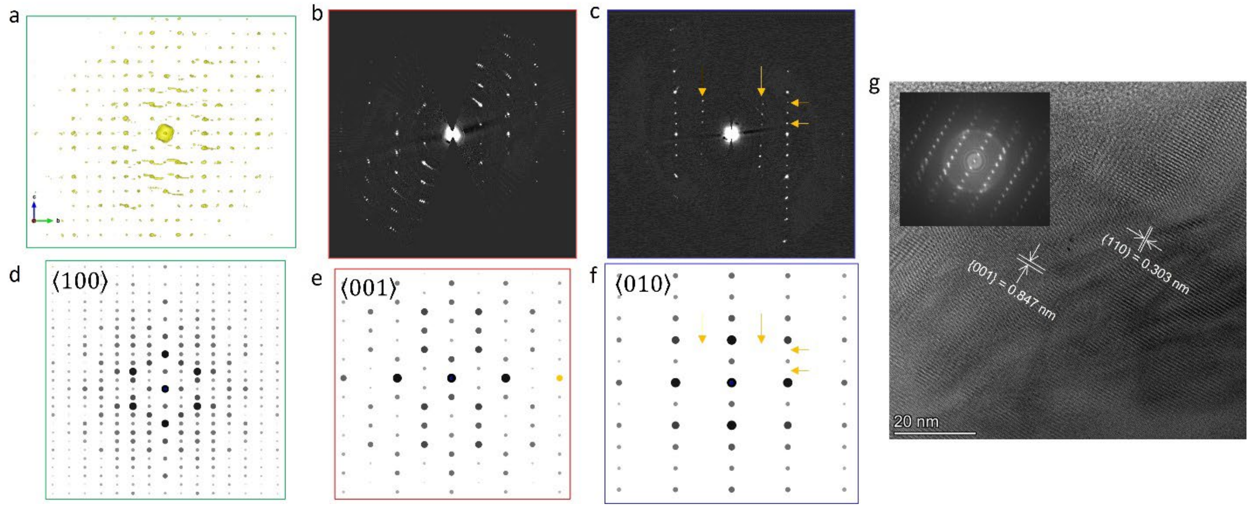

Figure S8. TEM analysis of Ga_3Rh phase based on sub-micrometer sized crystallites. a) 3D EDT data viewed along [100] direction (the [100] 2D slice does not contain enough information due to missing wedge in the tilt range). b) and c) 2D slice of the 3D EDT data along [001] and [010] respectively. (d) – (f) are electron diffraction patterns along [100], [001], and [010] axis, respectively, simulated using a structure model derived from the powder XRD. (g) an HRTEM image from a thin region of a crystallite projected close to [-110] direction (sample tilted about 10 degrees about the x-axis of the imaging plane). The image indicates tilting (i.e., waviness) of the (001) lattice planes, which is different from the rigid lattice in the Ga_9Rh_2 phase. Multiple occasions of such waviness of stacking were observed in small crystallites evidenced by either diffraction or imaging. Many kinematically forbidden reflections (in simulated [010] pattern) are present in experimental data as pointed out representatively by the yellow arrows in (c) and (f). This is likely because of the broken symmetry due to waviness of the stacking of the Ga-Rh antiprism cage along the [001] direction (edge-sharing direction).

Catalysis Studies

The catalytic studies were performed in a continuous flow laboratory setup (see Fig. S9). To test the two intermetallic compounds as catalysts in PDH reaction, the as-synthesized IMCs were ground to a fine powder (particle size around 20 μm, see Figure S15) in an agate mortar to increase the available surface area for reaction. The powder was weighed (202.3 mg of Ga_3Rh and 150.0 mg of Ga_9Rh_2) and mixed with silica support (~1.5 g) [9] in order to disperse the powder in a larger volume suitable for fixed bed catalytic studies. The IMC-support mixture was transferred into a tubular, fixed-bed quartz reactor (length: 650 mm; inner diameter: 10 mm). The quartz tube was placed in an electrically heated split furnace. The catalytic testing was conducted at 550 °C. The catalyst was first pre-treated in a flow of 20% hydrogen in helium for 3 h. After purging the reactor with helium for 1 h, the catalytic activity of the IMCs was tested in a flow of 8.9 mL_N min^{-1} propane (99.95% purity, Linde Gas) as feed gas diluted in 89 mL_N min^{-1} helium (99.996% purity, Linde Gas). The conversion and the selectivity during the 12 h on stream are shown in Fig. S10.

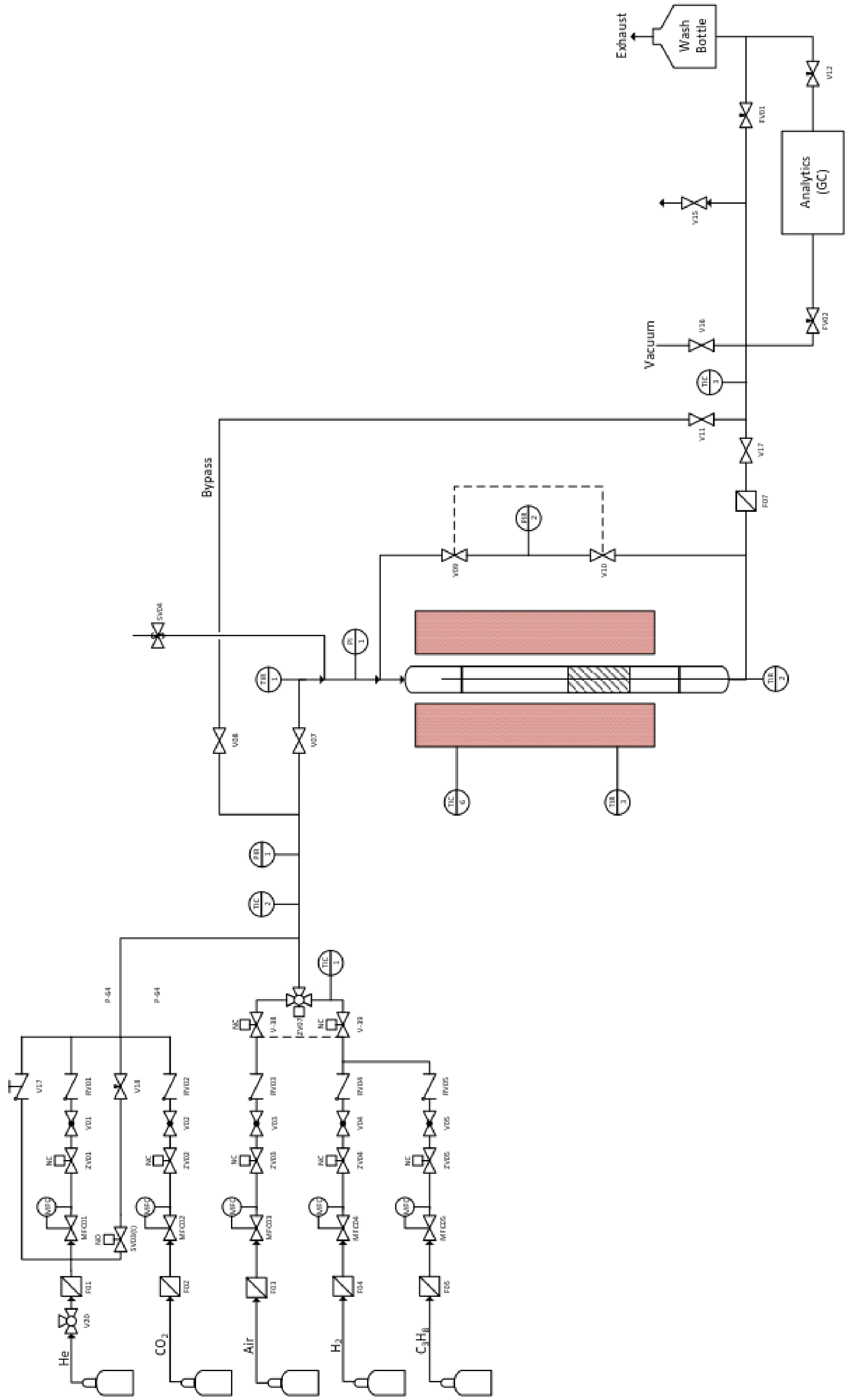

Figure S9. Flow scheme of the continuous gas-phase reactor used for propane dehydrogenation studies.

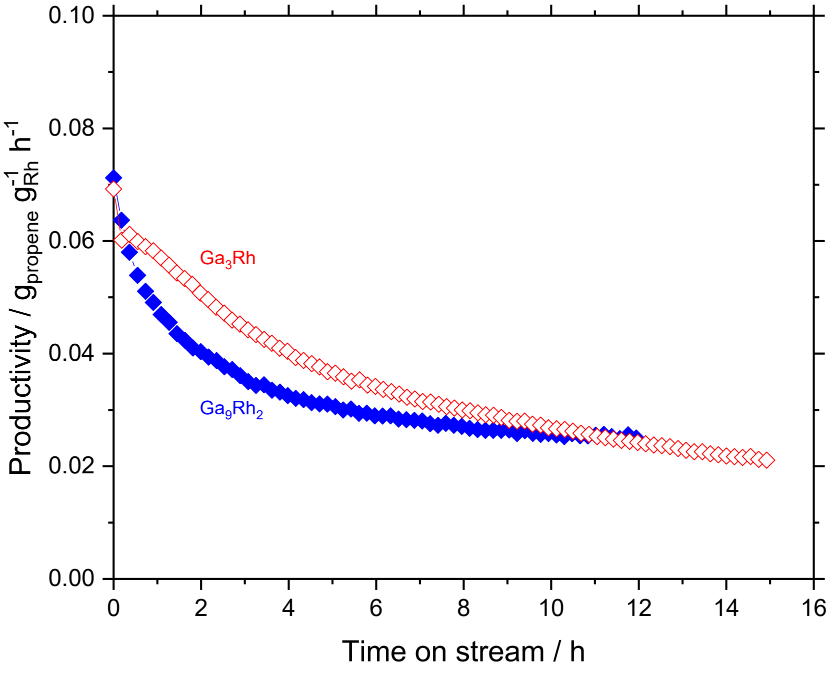

Figure S10. Productivity in propane dehydrogenation using Ga_3Rh (red) and Ga_9Rh_2 (blue) at 550 °C and 1.2 bar. Reaction conditions: Catalyst bed composition: 202.3 mg Ga_3Rh + 1.5 g silica; 150.0 mg Ga_9Rh_2 + 1.5 g silica, Gas flows: He flow 89 $mL_N\ min^{-1}$, C_3H_8 flow 8.9 $mL_N\ min^{-1}$.

$$Productivity = \frac{Amount\ of\ propane\ converted\ to\ propene(g.\ h^{-1})}{weight\ of\ Rh\ in\ bed\ (g)} \quad [g_{propene} g_{IMC\ or\ Rh}^{-1} h^{-1}]$$

XPS and HAXPES

Synchrotron Photoemission Studies:

Soft/Hard x-ray photoelectron spectroscopy (XPS/HAXPES) experiments were conducted at the SISSY-1 endstation located at the U17 and UE48 undulator beamline of the Energy Materials In-Situ Laboratory (EMIL) of Helmholtz-Zentrum-Berlin (HZB). The SISSY-1 setup houses a Scienta EW 4000 hemispherical electron analyzer that together with the two-color beamline of EMIL is capable to perform photoelectron spectroscopy in the soft and hard X-ray regime at UHV conditions (base pressure $< 2 \cdot 10^{-9}$ mbar). The soft X-rays are provided by the UE48 PGM undulator beamline, which delivers a high photon flux at a low spot size in the ~10 µm range over the energy range from 100 to 1500 eV. In addition, hard X-rays from 2000 to 10000 eV are provided by the U17 DCM undulator beamline, which is also focused in the SISSY-1 end-station at the same position on the sample. The endstation is connected through the UHV backbone in EMIL (sample transfer without breaking UHV conditions) with a vacuum chamber for sample annealing experiments with temperatures ranging from 30ºC to 1000ºC.

Prior to photoemission experiments the samples were transferred in air; therefore the surfaces of both samples contain significant amounts of gallium oxide. Gallium very readily forms Ga_2O_3 upon exposure to molecular oxygen or water. In vacuum and in the presence of metallic Ga, Ga_2O_3 can be decomposed to the volatile suboxide Ga_2O by heating up to 400°C.[10] This suboxide desorbs from the surface. The reaction can be used to create metallic Ga surfaces. We cleaned the samples by annealing at 600°C for 10 min. Fig. S11 and S12 summarize the changes in the XPS regions before and after annealing. It is however unclear, whether the observed Ga_2O_3 results from the surface decomposition of the intermetallic compound or Ga residues from the preparation.

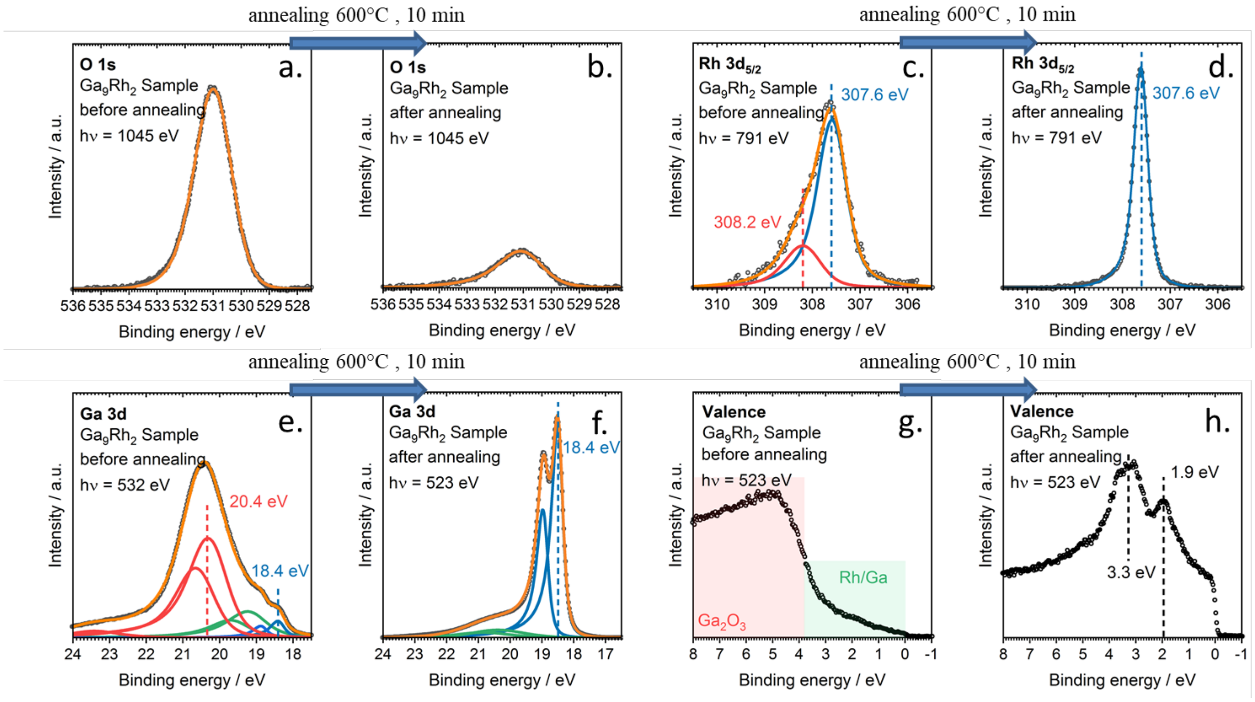

Figure S11: XPS spectra of the O 1s, Rh $3d_{5/2}$, Ga 3d, and valence regions for Ga_9Rh_2. Before annealing (a, c, e, g), we see significant amounts of Ga_2O_3, as is reflected in the O 1s region and the red components in the Ga 3d region. After annealing (b, d, f, h), the O 1s and the oxidic Ga 3d signals decrease significantly. This is also accompanied by significant changes in the Rh 3d and valence regions.

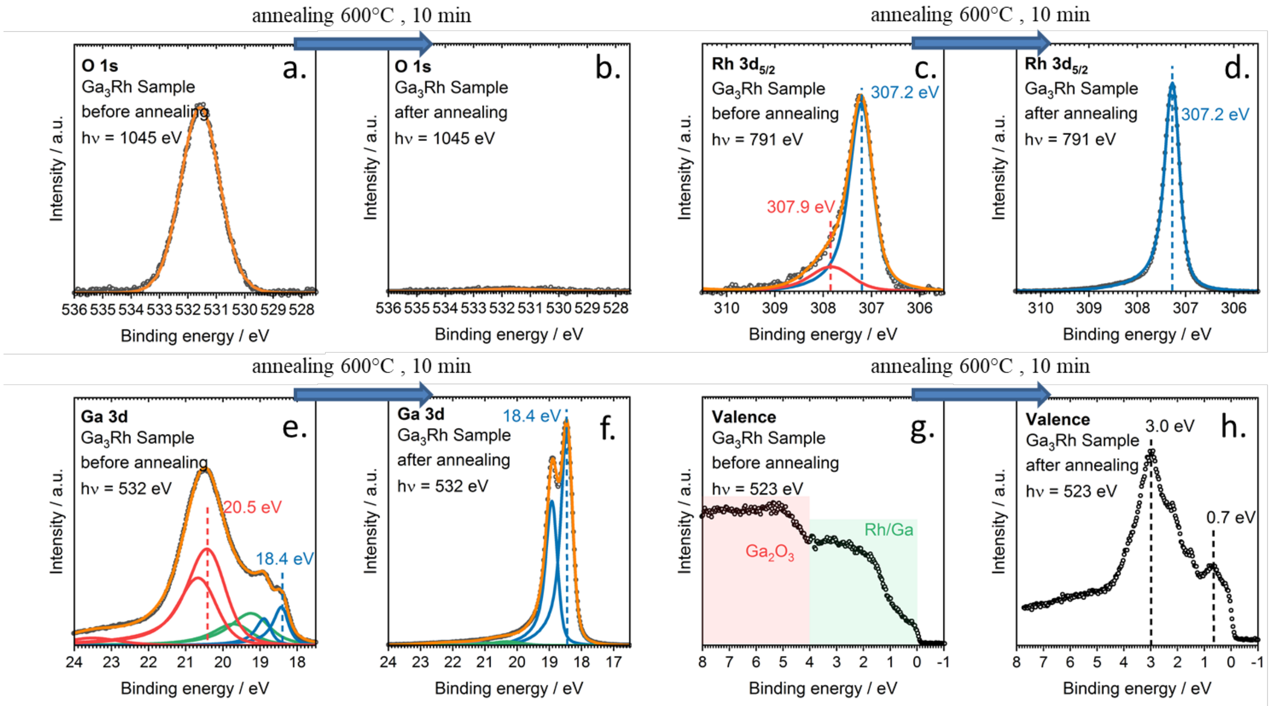

Figure S12: XPS spectra of the O 1s, Rh $3d_{5/2}$, Ga 3d, and Valence regions for Ga_3Rh. Before annealing (a, c, e, g), we see significant amounts of Ga_2O_3, as is reflected in the O 1s region and the red components in the Ga 3d region. After annealing (b, d, f, h), the O 1s and the oxidic Ga 3d signals decrease significantly. This is also accompanied by significant changes in the Rh 3d and valence regions.

Depth profiling:

The EMIL beamline allows for XPS measurements with soft and hard X-rays in the same setup. During our measurements, the hard X-ray undulator was still in commissioning; therefore we can only present hard X-ray results for the Ga_3Rh system but expect similar results for Ga_9Rh_2. We used hard X-rays 3740 eV for probing deeper into the bulk of the Ga_3Rh sample before annealing. HAXPES spectra of the Ga $2p_{1/2}$, O 1s, Rh $3d_{5/2}$, and Ga 3d components are shown in Fig. S13.

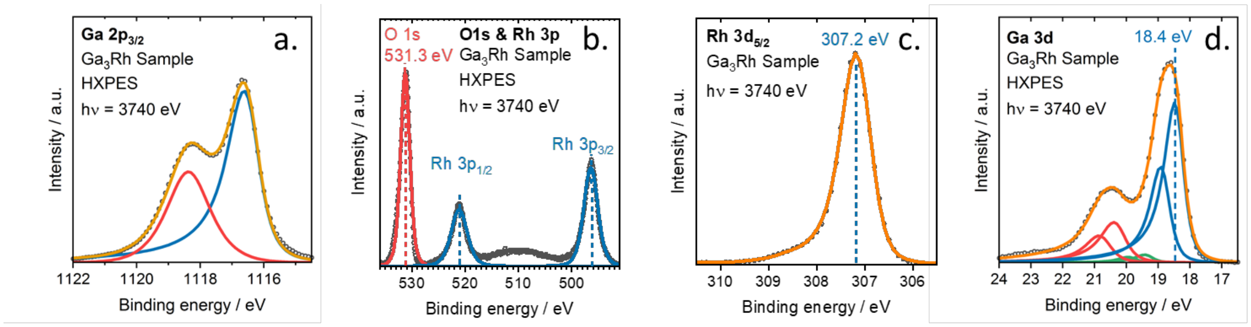

Figure S13: HAXPES spectra of the Ga $2p_{1/2}$, O 1s, Rh $3d_{5/2}$, and Ga 3d regions of the Ga3Rh sample before annealing.

We observe the same binding energies for the metallic Rh $3d_{5/2}$ and Ga 3d signals as in Fig. S12. Note that the higher excitation energy results in a significant increase of the metallic/oxidic signal ratio for Ga 3d from 0.18 to 2.91, this suggests that the oxidation is limited to the surface near region. This is further supported when comparing the metallic/oxidic signal ratio of the more surface sensitive Ga $2p_{1/2}$ signal 1.84 to the more bulk sensitive Ga 3d signal 2.91.

Quantification:

For quantification, the measured XPS spectra were fitted using CASAXPS 2.318. For quantification, we used photoemission cross-sections σ_i and asymmetry parameters β, δ, γ by Trzhaskovskaya, Nefedov, and Yarzhemsky.[11, 12] For linearly polarized light, the angular distribution of photoelectrons is given by

$$\frac{d\sigma_i}{d\Omega} = \frac{\sigma_i}{4\pi}[1 + \beta P_2(\cos\theta) + (\delta + \gamma\cos^2\theta)\sin\theta\cos\varphi]$$

The angles for the experimental geometry at the EMIL beamline are $\theta = 49°$ and $\varphi = 45°$. We approximate the photoemission cross-section as

$$\int_{\Omega'} \frac{d\sigma_i}{d\Omega} d\Omega \approx \frac{d\sigma_i}{d\Omega} \Omega'.$$

The surface Rh content is then calculated according to:

$$\frac{\frac{\mathrm{I_{Rh\ 3d}}}{\frac{\mathrm{d}\sigma_{\mathrm{Rh\ 3d}}}{\mathrm{d}\Omega}}}{\frac{\mathrm{I_{Rh\ 3d}}}{\frac{\mathrm{d}\sigma_{\mathrm{Rh\ 3d}}}{\mathrm{d}\Omega}} + \frac{\mathrm{I_{Ga\ 3d}}}{\frac{\mathrm{d}\sigma_{\mathrm{Ga\ 3d}}}{\mathrm{d}\Omega}}} * 100\ \mathrm{at.\%}.$$

Theory part:

Computational Details:

Density-functional theory (DFT) calculations were carried out using the *Vienna Ab Initio Simulation Package* (VASP) employing the projector augmented wave (PAW) method to represent the atomic cores and a plane wave basis set with a kinetic energy cutoff of 400 eV.[13-15] The functional developed by Perdew, Burke, and Ernzerhof (PBE) was applied to describe exchange-correlation effects.[16] A first-order Methfessel-Paxton smearing with a width of 0.2 eV was chosen for geometry optimizations and a tetrahedron smearing with Blöchl corrections for electronic density of states (DOS) calculations.[17, 18] Bader charges were evaluated using all-electron charge densities.[19, 20]

For Ga_3Rh a 20x7x8 Γ-centered k point mesh was used and for Ga_9Rh_2 a 10x10x8 Γ-centered k point mesh was chosen. The SCF convergence criterion was set to 10^{-8} eV. In geometry optimizations, atomic positions, as well as lattice constants, were allowed to relax with a force convergence criterion of 10^{-3} eV/Å.

The formation energy per atom is calculated as

$$E_{form}(Ga_xRh_{1-x}) = E(Ga_xRh_{1-x}) - xE(Ga) - (1-x)E(Rh)$$

with $E(Ga)$ and $E(Rh)$ as the energy of a Ga or Rh atom in its most stable modification.

Core level binding energies ε_{CL} for the Rh 3d core levels were evaluated in initial state approximation by calculating the Kohn-Sham eigenvalues of the respective core orbitals and in final-state approximation using the Janak-Slater transition state method by removing half an electron from the core orbital of interest and adding it to the Fermi level.[21, 22] The core level binding energy is then referenced to the Fermi level ε_F as $\varepsilon_{CL} = -(\varepsilon_C - \varepsilon_F)$ with the absolute core orbital energy ε_C. Furthermore, effects arising from the core electron screening are not accounted for in the calculations using the PAW approximation, which introduces considerable errors in absolute binding energies. Therefore, core level shifts (CLS) are calculated with respect to a pure Rh bulk reference ($\varepsilon_{CL,Rh}$) as $E_{CLS} = \varepsilon_{CL} - \varepsilon_{CL,Rh}$ to obtain relative shifts which are expected to yield accurate results. The center of the Rh 4d band ε_d was computed as $\varepsilon_d = \int x\rho(x)dx \, / \int \rho(x)dx$ with the electronic DOS distribution $\rho(x)$.

All inequivalent low-index surfaces with a maximum Miller index of 1 were generated using the Pymatgen Python library [23], in particular the pymatgen.core.surface module [24, 25] which is able to efficiently build unique surface slabs with different orientations and possible terminations. All atoms within 0.3 Å were considered as one layer. The thickness of the slab was ensured to be larger than 15 Å and a vacuum layer of over 15 Å was added. Additionally, a dipole correction was used to decouple periodic images in the direction perpendicular to the surface.[26]

The surface energy γ of a monometallic material can easily be computed from a slab with two equivalent surfaces via

$$\gamma = (E_{tot} - nE_{bulk})/2A$$

with the total energy of the relaxed slab E_{tot} and the bulk E_{bulk} as well as the number n of bulk unit cells in the slab. The factor of $1/2$ accounts for the presence of two surfaces in the slab model and A is the surface area. Unfortunately, for the calculation of surface energies of more sophisticated unit cells with different constituents as in the case of IMCs, it can appear that either the slab is nonstoichiometric or asymmetric. This hinders the straightforward application of the above equation for the surface energy. Therefore, we apply the method described in [27] where the surface energy is defined as

$$\gamma = (E_{cle} + E_{rel})/A$$

introducing the cleavage energy E_{cle} and the relaxation energy E_{rel}, respectively. The former is obtained by creating a stoichiometric, asymmetric slab with different terminations at the top and the bottom surface of the slab such that the two surfaces are complementary, i.e., if no vacuum would be added in z-direction the bulk structure would be obtained. The cleavage energy is distributed equally between the two surfaces as

$$E_{cle} = (E_{sp} - nE_{bulk})/2A$$

where E_{sp} is the single point energy of the unoptimized slab. The relaxation energy can then easily be obtained by relaxing the top / bottom part of the slab while keeping the bottom / top layers (half of the atoms) fixed and calculating

$$E_{rel} = (E_{relax} - E_{sp})/A.$$

Here, E_{relax} is the total energy of the slab with either the top or the bottom surface relaxed. Note that due to the huge amount of possibilities, reconstructed surfaces are not included in the present study.

Further computational results and discussion:

Table S4: Calculated lattice parameters and formation energies per atom for Ga_3Rh and Ga_9Rh_2 calculated with the PBE functional.

System	a / Å	b / Å	c / Å	β / °	E_{form} / eV
Ga_3Rh	3.2877	9.1993	8.4935	/	-0.47
Ga_9Rh_2	6.4736	6.4644	8.8767	93.240	-0.36

Table S5: Summary of the results regarding the electronic structure of Ga_3Rh and Ga_9Rh_2: Bader charge analysis, initial state (E^i_{CLS}) and final state (E^f_{CLS}) CLS with respect to a pure Rh bulk reference and the difference between the two. Additionally, the d-band shift with respect to pure Rh is given.

System	Charge / e	E^i_{CLS} / eV	E^f_{CLS} / eV	$E^f_{CLS} - E^i_{CLS}$ / eV	d-band shift / eV
Ga_3Rh	-0.83	-0.63	-0.12	+0.51	-0.02
Ga_9Rh_2	-0.93	-0.34	+0.22	+0.56	0.14

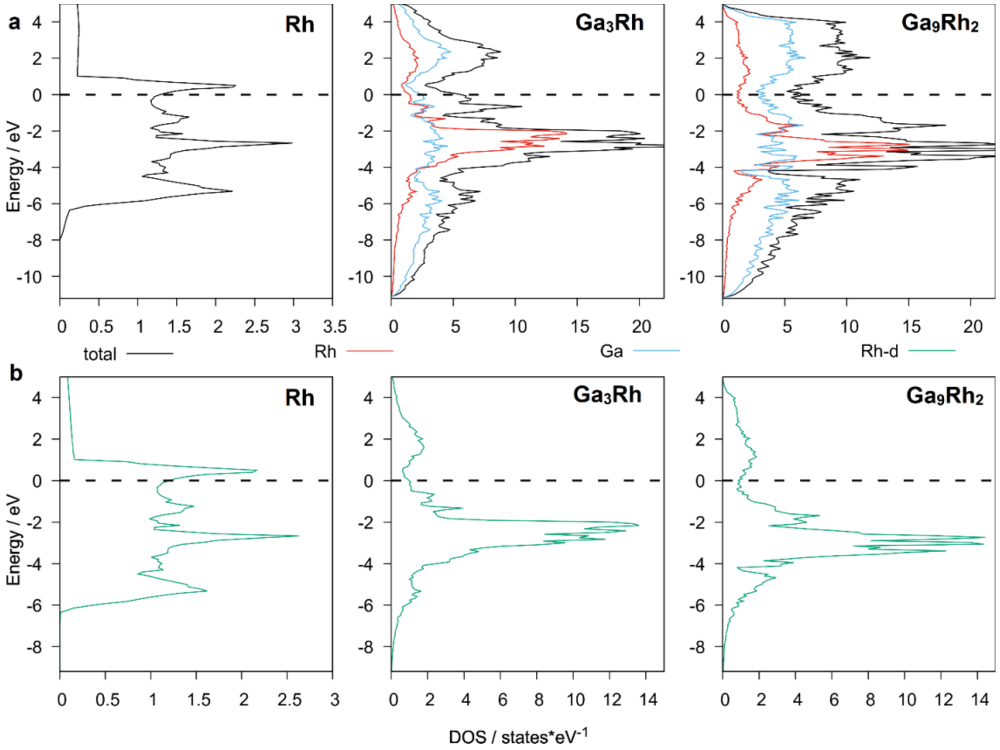

Figure S14: a) Total (black) DOS, Ga (blue) and Rh (red) contribution to the DOS as well as b) Rh 4d partial DOS (green) for Rh, Ga_3Rh, and Ga_9Rh_2 (from left to right). The Fermi level is indicated by a dashed line.

Besides a slight deviation (less than 2.5%) of the lattice constant, no structural changes occurred in the geometry optimization of the experimentally determined structures using DFT.

Bader charge calculations (see Table S6) reveal that Rh in Ga_9Rh_2 is slightly more negatively charged (by 0.1 e) than in Ga_3Rh. This can readily be explained by the geometric structure, i.e. by the higher Rh content in Ga_3Rh which results in a smaller nearest Rh-Rh distance ($d_{Rh\text{-}Rh}$ = 3.28 Å) compared to Ga_9Rh_2 ($d_{Rh\text{-}Rh}$ = 4.50 Å).

In order to compare with the XPS results, Rh-3d core-level shifts (CLS) of the two IMCs were calculated with respect to a pure Rh bulk reference in initial as well as final state approximation (see Table S6). As also described in the main manuscript for both compounds the CLS in final state approximation (E^f_{CLS}) is in excellent agreement with the experimental results (Ga_3Rh: -0.2 eV (exp.) and -0.12 eV (DFT); Ga_9Rh_2: +0.2 eV (exp.) and +0.22 eV (DFT)). However, these results are counterintuitive as the calculated Bader charge on the Rh atoms is negative, in the case of

Ga_9Rh_2 even more negative than for Ga_3Rh. This would lead to a negative core level shift, as found in the calculation using the initial state approximation (E^i_{CLS}). Interestingly, E^i_{CLS} is considerably smaller for Ga_9Rh_2 (-0.34 eV) than for Ga_3Rh (-0.63) even though the latter possesses a less negative Bader charge. This can be explained by examining the DOS of the alloys, more precisely, the Rh 4d band, which is plotted in Fig. S14b for bulk Rh, Ga_3Rh, and Ga_9Rh_2. It can be observed that, due to a higher filling of the Rh states, the d band shifts away from the Fermi level. Additionally, the band drastically changes its shape upon alloying. These two effects, the change in position and shape of the d band, lead to a shift of the d band center, which was shown to give an estimate for the initial state CLS for alloys studied in the literature.[28-30]

For Ga_3Rh, the d band center shift with -0.02 eV is smaller than for Ga_9Rh_2 with 0.14 eV, relative to the pure Rh metal, see Fig. S14. This also explains the less negative total initial state CLS of Ga_9Rh_2.

Finally, it remains to discuss final state effects, which include contributions from the core-hole relaxation to the core level binding energy. Therefore, we calculated the difference between initial and final state CLS ($E^f_{CLS} - E^i_{CLS}$, see Table S5) which is in a similar range for the two systems with +0.56 eV for Ga_9Rh_2 and +0.51 eV for Ga_3Rh compared to pure Rh. This is a known effect, which originates from a modified core-hole screening in the pure metal and the alloy which, in turn, is determined by the DOS at the Fermi level.[28, 29, 31-33] In the pure metal, contributions to the DOS at the Fermi level are mainly due to the Rh 4d orbitals, while in the alloys, the amount of d contribution to the DOS diminishes and the screening charge has more sp character increasing the final state effect.[28, 29] Overall, the smaller initial state shift together with a larger final state contribution lead to a positive shift of the Rh 3d core level binding energy in Ga_9Rh_2 compared to pure Rh, while the larger initial state shift and a slightly smaller final state contribution yield a negative CLS for Rh in Ga_3Rh.

Discussion of surface energies

The surface energies of all calculated surfaces as well as the cleavage energies are given in Table S6 and S7 for Ga_3Rh and Ga_9Rh_2, respectively. In total, the procedure described in the Computational Details section yields 22 different slab geometries in the case of Ga_3Rh and 71 geometries for Ga_9Rh_2, each producing two values for the surface energy, one for the top and one for the bottom surface. Please note that some of the slabs are symmetric and therefore less than 44 and 142 different surfaces exist for Ga_3Rh and Ga_9Rh_2, respectively. Obviously, the top and the bottom surface energies are equal in the case of a symmetric slab. The most stable surface directions and terminations are shown in Fig. 1 of the main text and confirm that Rh can be present as isolated atoms at the surface, that is without direct Rh nearest neighbors in the surface layer. From these most stable surfaces, only the (001) surface of Ga_3Rh consists of Rh atoms arranged in rows (with a Rh-Rh distance of 3.28 Å), instead of single isolated Rh atoms. For the other structures shown in Fig. 1 at least one Ga atom separates the surface Rh atoms. Due to the large number of different surface terminations (especially in the case of Ga_9Rh_2), it is not straightforward to make unambiguous predictions about the most favorable experimental surface termination. It is furthermore likely that this depends on the experimental preparation conditions.

Table S6: Surface energies (in J/m^2) of Ga_3Rh for all inequivalent low-index surfaces with different terminations. Besides the Miller index of the surface direction, the cleavage energy, as well as the surface energies of the top and the bottom surface are shown. For more information see the Computational Details section.

Miller Index	Number	E_{cleav}	γ_{top}	γ_{bottom}
(1 1 1)	1	1.07	0.94	0.94
	2	1.13	1.06	0.95
	3	1.32	1.26	1.03
	4	1.54	1.45	1.21
(1 1 0)	5	1.47	1.27	1.27
	6	1.25	1.12	1.05
	7	1.37	1.14	1.31
	8	1.27	1.07	1.07
(1 0 1)	9	1.44	1.13	1.13
	10	1.23	1.15	1.04
(1 0 0)	11	1.16	1.01	1.01
(0 1 1)	12	1.37	1.13	1.26
	13	1.35	1.15	1.28
	14	1.25	1.11	1.11
	15	1.63	1.48	1.35
	16	1.74	1.51	1.51
(0 1 0)	17	1.14	1.11	1.11
	18	1.29	1.19	1.24
	19	1.92	1.83	1.51
	20	1.52	1.34	1.34
(0 0 1)	21	1.19	1.09	1.09
	22	1.18	1.15	1.03

Table S7: Surface energies (in J/m^2) of Ga_9Rh_2 for all inequivalent low-index surfaces with different terminations. Besides the Miller index of the surface direction, the cleavage energy, as well as the surface energies of the top and the bottom surface are shown. For more information see the Computational Details section.

Miller Index	Number	E_{cleav}	$E_{surf,top}$	$E_{surf,bottom}$
(1 1 1)	1	1.14	0.70	0.96
	2	1.18	0.61	0.50
	3	1.04	0.69	0.69
	4	1.01	0.62	0.69
	5	0.92	0.77	0.89
	6	1.01	0.75	0.77
	7	0.99	0.98	1.05
	8	1.12	1.04	1.09
	9	1.22	1.20	0.98
	10	1.35	0.75	0.60
(1 1 0)	11	1.22	1.00	0.91
	12	1.32	1.07	0.90
	13	1.33	1.06	1.13
	14	1.42	0.93	0.91
	15	1.30	1.12	1.01
	16	0.98	0.75	0.76
	17	1.00	0.67	0.86
	18	0.97	0.66	0.73
	19	1.18	0.93	0.73
(1 1 -1)	20	1.20	0.80	1.00
	21	1.15	0.75	0.92
	22	1.17	0.97	0.87
	23	1.06	0.85	0.79
	24	1.03	0.79	0.80
	25	1.25	1.06	0.94
	26	1.26	0.93	1.04
	27	1.37	0.88	0.97
	28	1.34	0.98	1.01
	29	1.35	1.06	0.93
	30	1.28	0.99	1.00
	31	1.19	0.95	0.91
(1 0 1)	32	1.06	0.89	0.77
	33	1.21	0.72	1.01
	34	1.15	0.85	0.95
	35	1.11	0.89	0.89
(1 0 0)	36	1.03	0.88	0.75
	37	1.16	0.83	0.96
	38	1.51	1.20	1.23
	39	1.26	0.93	1.16

	40	1.25	0.83	1.09
	41	1.08	0.84	0.92
(1 0 -1)	42	1.24	0.91	0.93
	43	1.25	0.79	1.01
	44	1.17	0.77	0.97
	45	1.12	0.95	0.95
	46	1.11	1.00	0.88
	47	1.18	0.89	0.92
	48	1.34	1.11	0.91
(0 1 1)	49	1.20	0.94	0.89
	50	1.17	0.97	0.87
	51	1.18	1.04	0.88
	52	1.29	1.05	1.14
	53	1.30	1.08	1.11
	54	1.21	1.05	0.96
	55	1.12	0.88	0.88
	56	1.01	0.81	0.78
	57	1.10	0.87	0.78
	58	1.16	0.90	0.78
(0 1 0)	59	1.21	0.92	0.92
	60	1.14	0.90	0.93
	61	1.18	0.98	0.95
	62	1.27	0.85	1.09
	63	1.18	0.91	0.91
	64	1.22	1.03	0.77
(0 0 1)	65	1.18	0.98	0.94
	66	1.16	1.03	0.79
	67	1.07	0.92	0.71
	68	1.27	0.96	1.15
	69	1.23	1.01	1.08
	70	1.16	0.96	0.99
	71	1.18	0.96	0.93

SEM Data of the particles used in the catalytic tests

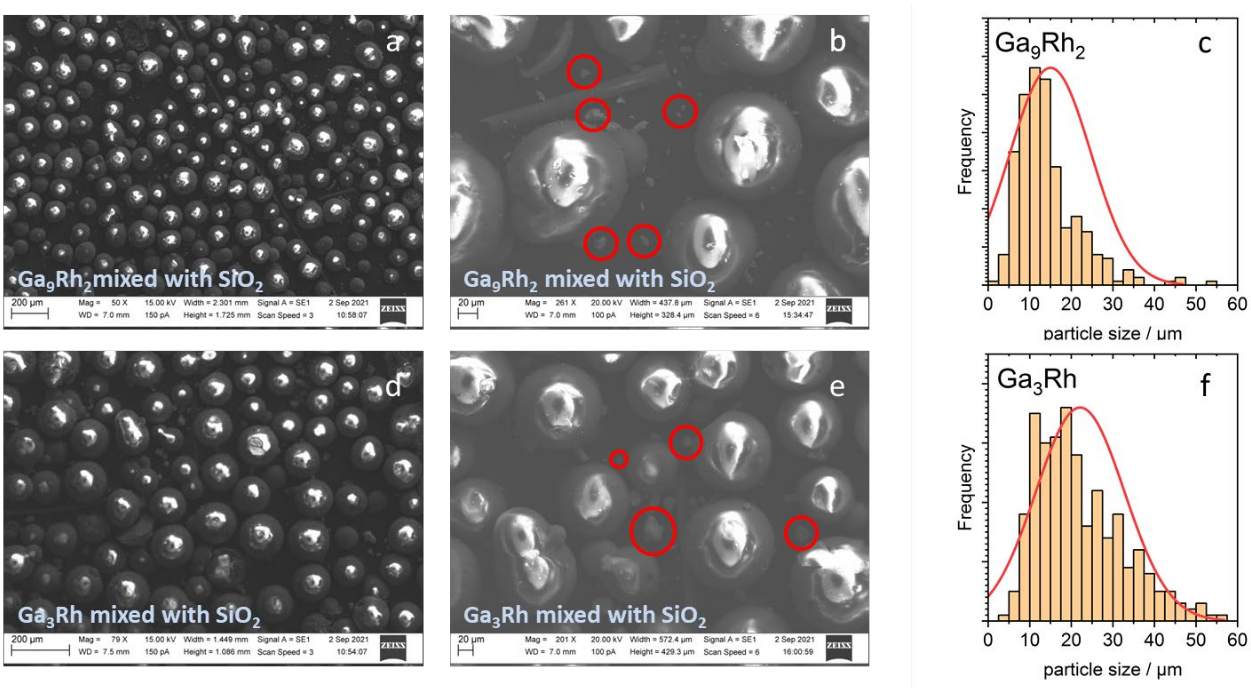

Figure S 15: SEM micrographs of the samples used in the catalytic tests for (a,b) Ga_9Rh_2 and (d,e) Ga_3Rh. Shown is the mixture used in the catalytic tests, the large spheres are SiO_2 particles, scattered between these are the milled particles of the intermetallic compounds (red circles in (b, e)). Based on a total of 10 micrographs in the 50x magnification for each IMC powder the particle size was determined. The resulting particle size distributions are shown in panels c and e.

XRD before and after catalytic tests:

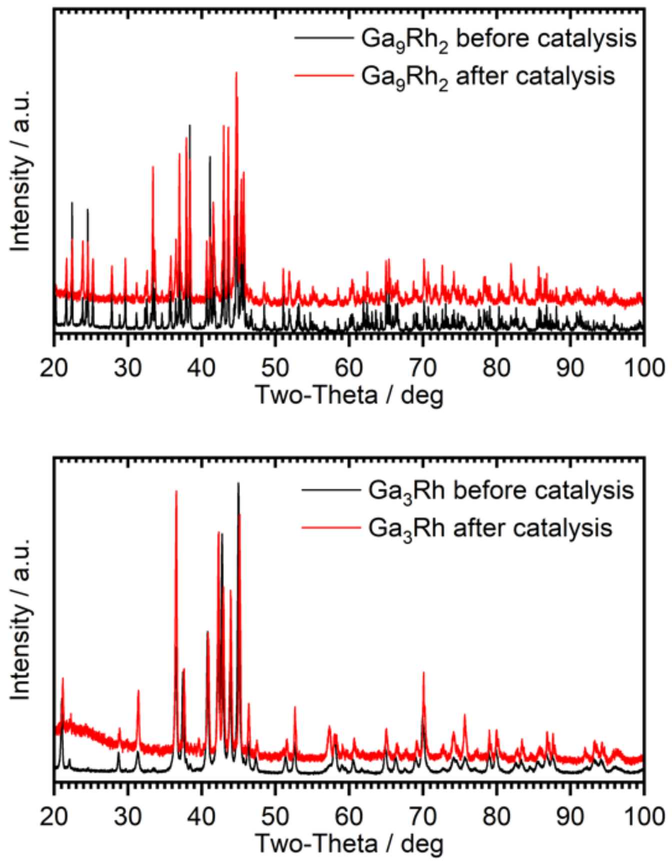

Figure S 16: XRD diffractograms of Ga_3Rh and Ga_9Rh_2 before and after catalytic tests. In both cases the reflex positions before and after catalysis agree, which shows that the bulk structure of the particles does not change during the test reaction.

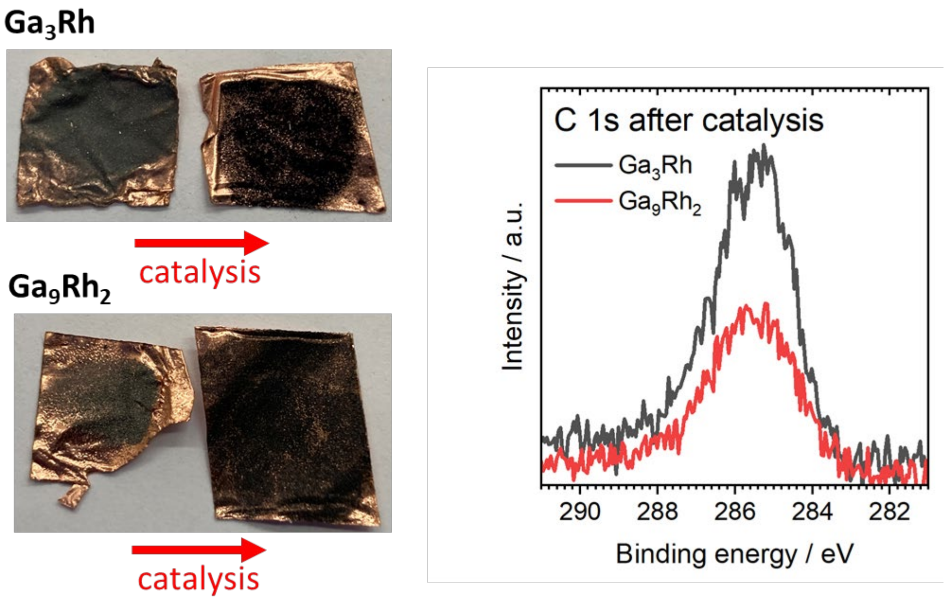

Figure S 17: Photographs and C 1s XP spectra of Ga_3Rh and Ga_9Rh_2 samples after catalytic testing. The XPS analysis shows significant C 1s signals corresponding to an increase in carbon surface concentration by about 20 at.% for both samples.

Literature:

[1] M. Bostrom, H. Rosner, Y. Prots, U. Burkhardt, Y. Grin, The Co2Al9 structure type revisited, Z. Anorg. Allg. Chem., 631 (2005) 534-541.
[2] K. Schubert, H. Breimer, R. Gohle, H.L. Lukas, H.G. Meissner, E. Stolz, Einige strukturelle Ergebnisse an metallischen Phasen III, 45 (1958) 360-361.
[3] K. Schubert, Zum Aufbau der Systeme Co-Ga, Pd-Ga, Pd-Sn und verwandter Legierungen, Z. Metallkunde, DOI (1959) 534.
[4] Materials, Data, Inc.
[5] International Tables for Crystallography: Space-group symmetry, 2 ed., International Union of Crystallography, Chester, England, 2016.
[6] C.I. GbR, Endeavour 1.5.
[7] H. Putz, J.C. Schon, M. Jansen, Combined method for ab initio structure solution from powder diffraction data, J. Appl. Crystallogr., 32 (1999) 864-870.
[8] L. Lutterotti, M. Bortolotti, Object Oriented Programming and Fast Computation Techniques in MAUD, a Program for Powder Diffraction Analysis Written in Java, Compcomm. Newslett., 1 (2003) 43-50.
[9] N. Raman, S. Maisel, M. Grabau, N. Taccardi, J. Debuschewitz, M. Wolf, H. Wittkämper, T. Bauer, M. Wu, M. Haumann, C. Papp, A. Görling, E. Spiecker, J. Libuda, H.P. Steinrück, P. Wasserscheid, Highly Effective Propane Dehydrogenation Using Ga-Rh Supported Catalytically Active Liquid Metal Solutions, ACS Catal., 9 (2019) 9499-9507.
[10] R. Carli, C.L. Bianchi, Xps Analysis of Gallium Oxides, Appl. Surf. Sci., 74 (1994) 99-102.
[11] M.B. Trzhaskovskaya, V.I. Nefedov, V.G. Yarzhemsky, Photoelectron angular distribution parameters for elements Z=1 to Z=54 in the photoelectron energy range 100-5000 eV, Atom. Data Nucl. Data, 77 (2001) 97-159.
[12] M.B. Trzhaskovskaya, V.I. Nefedov, V.G. Yarzhemsky, Photoelectron angular distribution parameters for elements Z=55 to Z=100 in the photoelectron energy range 100-5000 eV, Atom. Data Nucl. Data, 82 (2002) 257-311.
[13] G. Kresse, J. Furthmuller, Efficient iterative schemes for ab initio total-energy calculations using a plane-wave basis set, Phys. Rev. B, 54 (1996) 11169-11186.
[14] G. Kresse, J. Furthmuller, Efficiency of ab-initio total energy calculations for metals and semiconductors using a plane-wave basis set, Comp. Mater. Sci., 6 (1996) 15-50.
[15] G. Kresse, D. Joubert, From ultrasoft pseudopotentials to the projector augmented-wave method, Phys. Rev. B, 59 (1999) 1758-1775.
[16] J.P. Perdew, K. Burke, M. Ernzerhof, Generalized Gradient Approximation Made Simple, Phys. Rev. Lett., 77 (1996) 3865-3868.
[17] M. Methfessel, A.T. Paxton, High-precision sampling for Brillouin-zone integration in metals, Phys. Rev. B, 40 (1989) 3616-3621.
[18] P.E. Blochl, O. Jepsen, O.K. Andersen, Improved Tetrahedron Method for Brillouin-Zone Integrations, Phys. Rev. B, 49 (1994) 16223-16233.
[19] G. Henkelman, A. Arnaldsson, H. Jonsson, A fast and robust algorithm for Bader decomposition of charge density, Comp. Mater. Sci., 36 (2006) 354-360.
[20] W. Tang, E. Sanville, G. Henkelman, A grid-based Bader analysis algorithm without lattice bias, J. Phys. Condens. Mat., 21 (2009).
[21] J.F. Janak, Proof That Delta-E-Delta-Ni=Epsilon-I in Density-Functional Theory, Phys. Rev. B, 18 (1978) 7165-7168.
[22] J.C. Slater, Statistical Exchange-Correlation in the Self-Consistent Field, in: P.-O. Löwdin (Ed.) Advances in Quantum Chemistry, Academic Press1972, pp. 1-92.

[23] S.P. Ong, W.D. Richards, A. Jain, G. Hautier, M. Kocher, S. Cholia, D. Gunter, V.L. Chevrier, K.A. Persson, G. Ceder, Python Materials Genomics (pymatgen): A robust, open-source python library for materials analysis, Comp. Mater. Sci., 68 (2013) 314-319.
[24] W. Sun, G. Ceder, Efficient creation and convergence of surface slabs, Surf. Sci., 617 (2013) 53-59.
[25] F.A. Rasmussen, K.S. Thygesen, Computational 2D Materials Database: Electronic Structure of Transition-Metal Dichalcogenides and Oxides, J. Phys. Chem. C, 119 (2015) 13169-13183.
[26] J. Neugebauer, M. Scheffler, Adsorbate-substrate and adsorbate-adsorbate interactions of Na and K adlayers on Al(111), Phys. Rev. B, 46 (1992) 16067-16080.
[27] X. Tian, T. Wang, L. Fan, Y. Wang, H. Lu, Y. Mu, A DFT based method for calculating the surface energies of asymmetric MoP facets, Appl. Surf. Sci., 427 (2018) 357-362.
[28] I.A. Abrikosov, W. Olovsson, B. Johansson, Valence-band hybridization and core level shifts in random Ag-Pd alloys, Phys. Rev. Lett., 87 (2001).
[29] C. Hartwig, K. Schweinar, T.E. Jones, S. Beeg, F.P. Schmidt, R. Schlogl, M. Greiner, Isolated Pd atoms in a silver matrix: Spectroscopic and chemical properties, J. Chem. Phys., 154 (2021).
[30] W. Olovsson, C. Goransson, L.V. Pourovskii, B. Johansson, I.A. Abrikosov, Core-level shifts in fcc random alloys: A first-principles approach - art. no. 065203, Phys. Rev. B, 72 (2005).
[31] S.M. Haiko Wittkämper, Mingjian Wu, Johannes Frisch, Regan G. Wilks, Mathias Grabau, Erdmann Spiecker, Marcus Bär, Andreas Görling, Hans-Peter Steinrück, Christian Papp, Oxidation induced restructuring of Rh–GaSCALMS model catalyst systems, 153 (2020).
[32] H. Wittkamper, S. Maisel, M. Moritz, M. Grabau, A. Gorling, H.P. Steinruck, C. Papp, Surface oxidation-induced restructuring of liquid Pd-Ga SCALMS model catalysts, Phys. Chem. Chem. Phys., DOI 10.1039/d1cp02458b(2021).
[33] M. Armbruster, R. Schlogl, Y. Grin, Intermetallic compounds in heterogeneous catalysis-a quickly developing field, Sci. Technol. Adv. Mat., 15 (2014).
